# Potassium is a key signal in host-microbiome dysbiosis in periodontitis

**DOI:** 10.1371/journal.ppat.1006457

**Published:** 2017-06-20

**Authors:** Susan Yost, Ana E. Duran-Pinedo, Keerthana Krishnan, Jorge Frias-Lopez

**Affiliations:** 1 The Forsyth Institute, Cambridge, Massachusetts, United States of America; 2 Department of Oral Biology, University of Florida, Gainesville, Florida, United States of America; The University of Texas at Austin, UNITED STATES

## Abstract

Dysbiosis, or the imbalance in the structural and/or functional properties of the microbiome, is at the origin of important infectious inflammatory diseases such as inflammatory bowel disease (IBD) and periodontal disease. Periodontitis is a polymicrobial inflammatory disease that affects a large proportion of the world's population and has been associated with a wide variety of systemic health conditions, such as diabetes, cardiovascular and respiratory diseases. Dysbiosis has been identified as a key element in the development of the disease. However, the precise mechanisms and environmental signals that lead to the initiation of dysbiosis in the human microbiome are largely unknown. In a series of previous *in vivo* studies using metatranscriptomic analysis of periodontitis and its progression we identified several functional signatures that were highly associated with the disease. Among them, potassium ion transport appeared to be key in the process of pathogenesis. To confirm its importance we performed a series of *in vitro* experiments, in which we demonstrated that potassium levels a increased the virulence of the oral community as a whole and at the same time altering the immune response of gingival epithelium, increasing the production of TNF-α and reducing the expression of IL-6 and the antimicrobial peptide human β-defensin 3 (hBD-3). These results indicate that levels of potassium in the periodontal pocket could be an important element in of dysbiosis in the oral microbiome. They are a starting point for the identification of key environmental signals that modify the behavior of the oral microbiome from a symbiotic community to a dysbiotic one.

## Author summary

Homeostasis of the human microbiome plays a key role in maintaining the healthy status of the human body. Changes in composition and function of the human microbiome (dysbiosis) are at the origin of important infectious inflammatory diseases such as inflammatory bowel disease (IBD) and periodontal disease. However, the environmental elements that trigger the development of dysbiotic diseases are largely unknown. In previous studies, using community-wide transcriptome analysis, we identified ion potassium transport as one of the most important functions in the pathogenesis of periodontitis and its progression. Here, we confirm with a series of *in vitro* experiments that potassium can act as an important signal in the dysbiotic process inducing pathogenesis in the oral microbiome and altering the host response in front of the microbial challenge that could lead to microbial immune subversion. Our study provides new insights into the important role that ion potassium plays a signal in oral dysbiosis during periodontitis.

## Introduction

Dysbiosis, or the imbalance in the structural and/or functional properties of the microbiome, leads to the breakdown of host-microbe homeostasis, and has been associated with the pathogenesis of several important inflammatory diseases mediated by the activity of the microbial community, such as inflammatory bowel diseases (IBDs) [1,2] and periodontal diseases [3].

Periodontitis is a polymicrobial disease caused by the coordinated action of a complex microbial community, leading to inflammation and periods of active destruction of the tissues supporting the teeth. Periodontal inflammation has adverse impacts on a wide variety of systemic health conditions, such as diabetes, cardiovascular and respiratory diseases [4,5]. It is the sixth most prevalent disabling health condition in the world affecting approximately 750 million people worldwide [6]. It has been postulated that changes in the composition of subgingival biofilms could explain these periods of disease activity. In fact, a few studies have found differences in the levels of subgingival species when comparing progressing and non-progressing sites [7,8]. These studies also demonstrated considerable overlap in the composition of the microbial communities associated with progressing and non-progressing lesions, suggesting that the difference in the periodontal status of the sites could not be explained solely by differences in subgingival microbial composition. Recently, it has been proposed that certain organisms could act as 'keystone-pathogens' that modulate the behavior of the oral microbial community, which becomes dysbiotic [9]. Indeed, we have previously reported that organisms not considered pathogens express large numbers of putative virulence factors during chronic severe periodontitis and disease progression [10,11]. However, the environmental signals that trigger this change in behavior of the community remain for the most part unknown.

In two previous studies focused on the oral microbial metatranscriptome in health, disease and during periodontitis progression, gene ontology (GO) enrichment analysis showed that potassium ion transport was a key signature of microbial metabolic activities associated with disease [10,11]. In the present study we focus our interest on confirming our previous *in vivo* observations [11] on the potential role that potassium ion has as a signal that initiates changes in the oral microbiome, leading to dysbiosis of the microbial community. Potassium is the most abundant monovalent ion inside the cells. However, in healthy periodontal tissues potassium is present at low concentrations in the gingival crevicular fluid in contact with the oral biofilm [12–14]. A positive and statistically significant correlation has been found between the concentration of potassium in crevicular fluid and mean pocket depths [12], probably due to cell lysis of host cells. Here we performed a series of experiments to test the effects of potassium on plaque community gene expression, virulence, and inflammation and showed that levels of potassium ion act as an important environmental signal for microbial dysbiosis and epithelial response to the microbial challenge.

## Results

### Potassium alters the composition of the active oral microbiome

Potassium ion (K^+^) transport has previously been identified as an important signature among the metabolic activities of the oral microbiome in periodontitis [10,11]. However, the exact mechanisms by which potassium exerts its activity as an environmental signal leading to microbial dysbiosis remain unknown. To test the effects of potassium on plaque community gene expression, plaque was collected from a healthy human volunteer, exposed to saliva with or without added potassium, and subjected to metatranscriptomic analysis. We used K^+^ concentrations akin to those found in gingival crevicular fluid in severe periodontitis [13,14]. After only 3 hours of incubation RNA was extracted for analysis. We thus identified the initial reaction of the community to higher levels of K^+^ in the environment. We detected between 73.7 and 96.2% of all genes in our libraries, which represents a high sequencing depth across all samples (S1 Fig).

**Fig 1:**
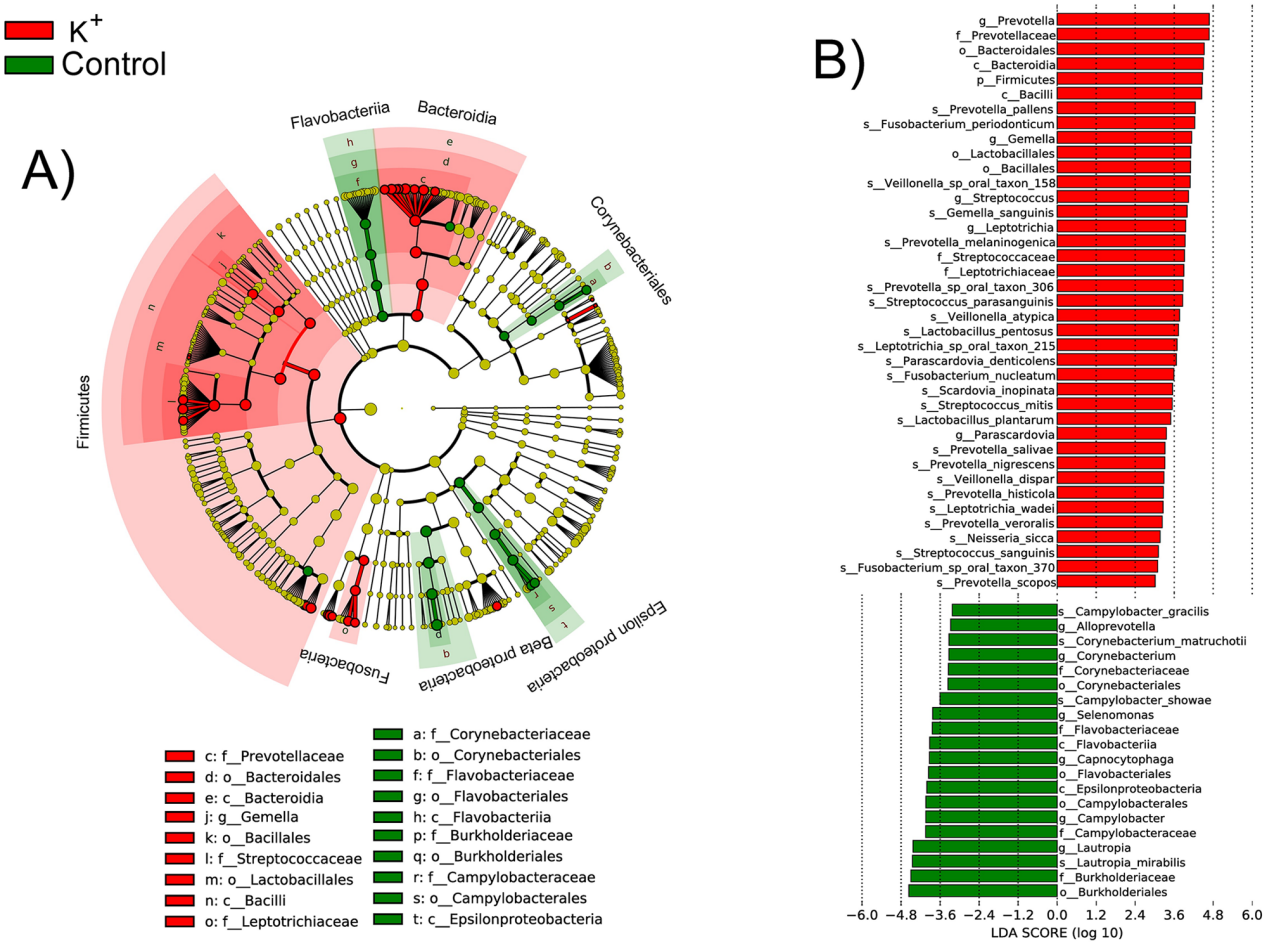
Statistical differences in metatranscriptome composition after addition of ion potassium. Phylogenetic assignment of the mRNA hits was performed using Kraken [17] and were analyzed using LEfSe with default parameters (p-value < 0.05 for Kruskal-Wallis rank sum test on classes and pairwise Wilcoxon test between subclasses of different classes) to identify significant differences in transcription activity at species level between the microbial communities compared. A) Cladogram showing the taxonomic distribution of lineages whose expression levels had a LDA value of 3.0 or higher as determined by LEfSe [18]. B) Histogram of LDA scores for differentially active taxa. In red are taxa whose activity, as determined by number of transcripts, was increased in the presence of K^+^. In green are species whose activity was higher in the absence of K^+^.

Phylogenetic assignment of the transcripts showed that several members of the community responded immediately to the presence of K^+^ in their surroundings (Fig 1). Transcripts were assigned to different taxa using Kraken and LEfSe was used to determine differentially transcriptionally active taxa. Among those that contribute a significantly higher fraction of transcripts to the metatranscriptome, we found organisms that previously have been associated with periodontal disease such as *Leptotrichia* spp., *Campylobacter* spp. and *Fusobacterium* spp. and *Prevotella* spp. [15], but also organisms that have been considered to be associated with health such as *Streptococcus* spp. Nonetheless *Streptococcus* spp. have been also found in large numbers in periodontal disease [15], and we previously identified them as producing large numbers of putative virulence factors at early states of dysbiosis during periodontitis progression [11]. Genus *Lautropia*, which has been associated with health [16], was significantly less active in the presence of K^+^. However, other groups of microorganisms that have been considered to be associated with periodontal disease such as *Corynebacterium* and *Campylobacter* [15,16] were less active in the presence of high concentration of K^+^.

### The oral microbiome becomes more virulent in the presence of ion potassium

**Fig 2:**
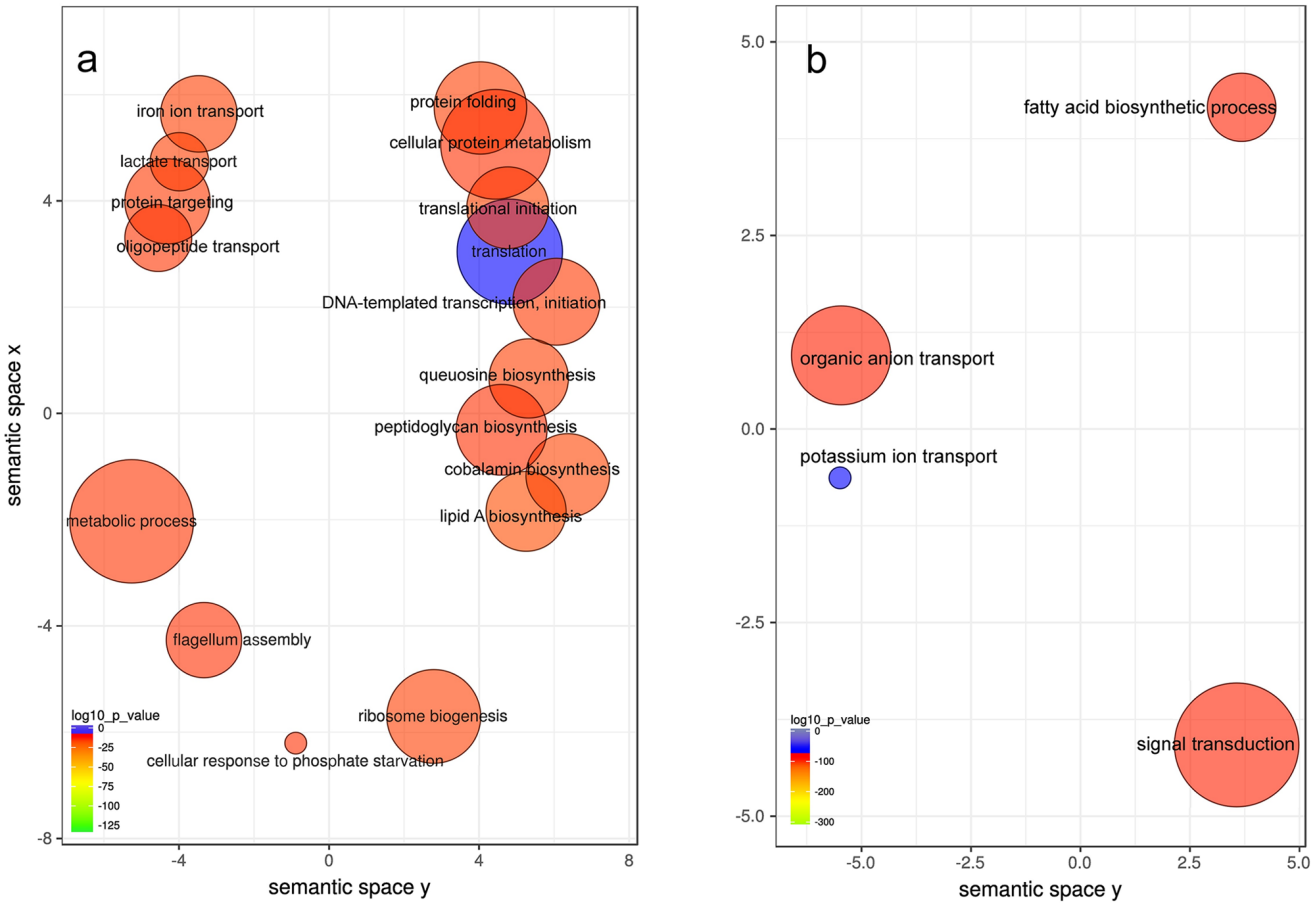
GO enrichment analysis comparing plaque response to the presence and absence of added ion potassium to the medium. Enriched terms obtained using GOseq were summarized and visualized as a scatter plot using REVIGO. Only GO terms with FDR adjusted p-value < 0.05 in the 'GOseq' analysis were used. A) Summarized GO terms related to biological processes after addition of K^+^. B) Summarized GO terms related to biological processes with no K^+^ added. Circle size is proportional to the frequency of the GO terms, color indicates the log10 p-value (red higher, blue lower). Distance between circles represent GO terms' semantic similarities. Each of the circles represent a GO term, which depending on the similarity in the terms included in them they will be closer or more distant in the graph.

To determine changes in metabolic activities in the whole community due to the increase in K^+^ concentration, we performed GO terms enrichment analysis. In order to mimic as much as possible the conditions present in the periodontal pocket during disease, we utilized levels of K^+^ of the same order as the ones found in severe periodontitis [12–14]. After only 3 hours of incubation, we observed at the community-wide level an over-representation of activities associated with disease, such as iron ion transport, oligopeptide transport, flagellum assembly and cobalamin biosynthesis (Fig 2a).

Among the GO molecular functions over-represented in the presence of K^+^ there are some linked to proteolysis, we found metallo-exopeptidase activity and aminopeptidase activity (S1 Table). Protease activity is a well established player in the pathogenesis of periodontal disease [19,20]. Consistent with low overall environmental levels of K^+^ in the environment, we observed an over-representation of potassium transport activities in the oral microbiome incubated without K^+^ added (Fig 2b, S1 Table).

We next determined whether the addition of K^+^ increased the synthesis of putative virulence factors. As a model we used hemolysins, which are recognized as potential virulence factors in a large number of anaerobic species [21].

**Fig 3:**
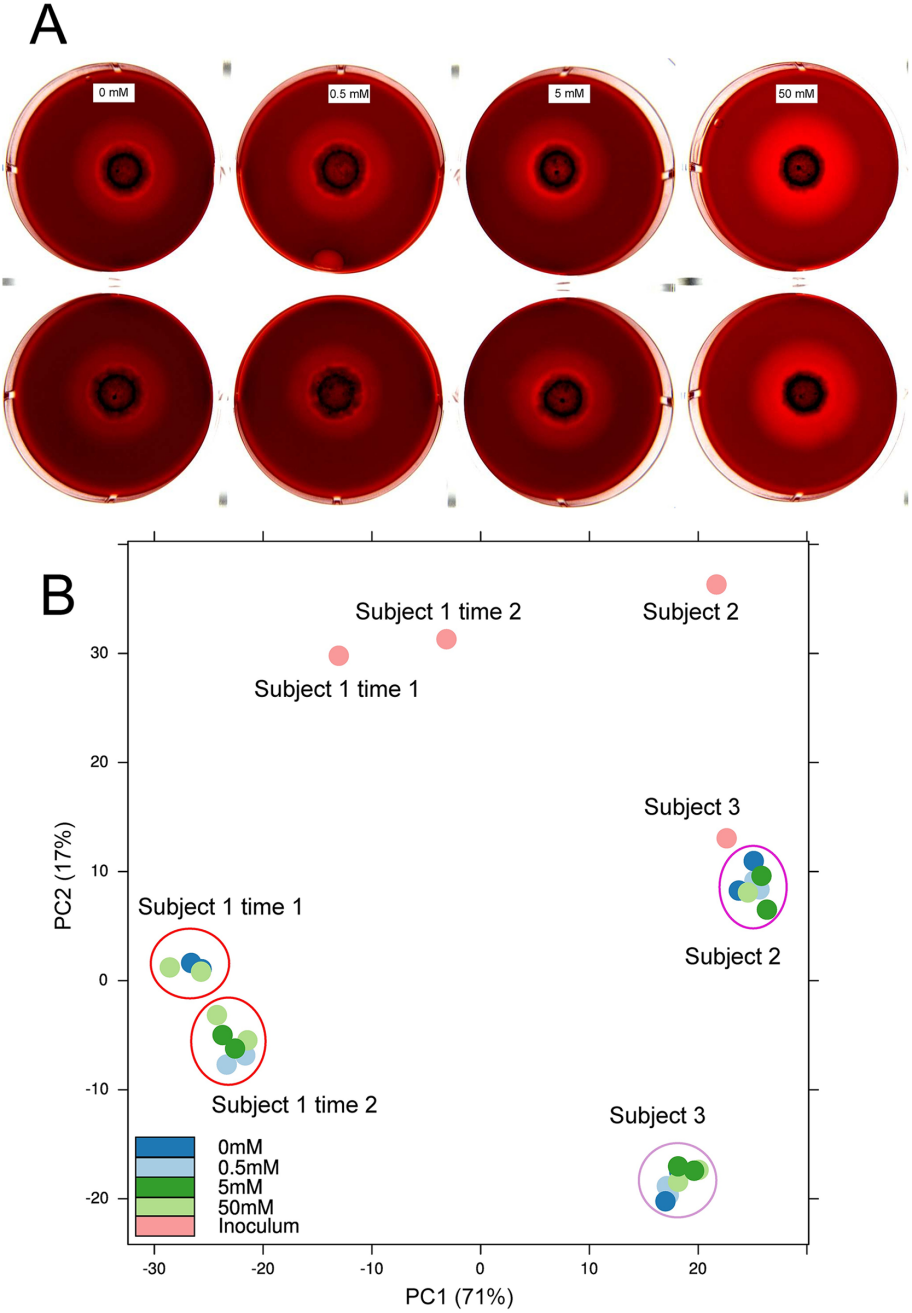
Effect of potassium concentration on hemolytic activity on dental plaque growing on blood agar plates. Hemolysis was assayed on horse blood agar plates with different concentrations of K^+^ added and incubated under anaerobic conditions at 37°C. A) Hemolytic activity of whole dental plaque on different concentrations of K^+^ added after 6 days of incubation in 2 different biological experiments. B) Principal component analysis of HOMINGs phylogenetic profiles of the final microbial communities that grew on blood agar plates from 3 different healthy individuals, the initial inocula. Subject 1 was analyzed at 2 different times (see Fig 3a).

In the case of whole dental plaque growing on plates with added K^+^, after 6 days incubation we observed hemolysis at all concentrations, with the 50mM concentration showing higher hemolytic activity, while lower concentrations and the control with no K^+^ added behaved similarly (Fig 3a). To show that the increase in hemolytic activity of the whole plaque at 50mM was due to changes in metabolic activities and not to changes in community composition, we characterized the microbial communities from the final plaque growing at the different concentrations of K^+^ from 3 different subjects (Fig 3b). The results of the phylogenetic composition based on 16s rRNA deep sequencing of those communities showed no differences between the communities growing at different concentrations of K^+^ and the control (Fig 3b and S2 Table). Additionally, we characterized the composition of the initial inoculum used in the experiments and the microbial communities from a periodontally healthy and from a sample with severe periodontitis. The initial inocula for all three patients analyzed was identical in total number of cells and after 6 days of incubation all plates from a single experiment had the same number of total cells growing (S3 Table). The microbial composition of the original inoculum from the different subjects was different as well as the final composition of the communities growing on plates (Fig 3b). However, those communities showed no differences between the communities growing at different concentrations of K^+^. Collectively, these results indicate that an increase in K^+^ in the environment leads to expression of genes associated with pathogenicity in the oral microbiome, which could be important in the process of dysbiosis, from commensal to pathogenic microbiome.

**Fig 4:**
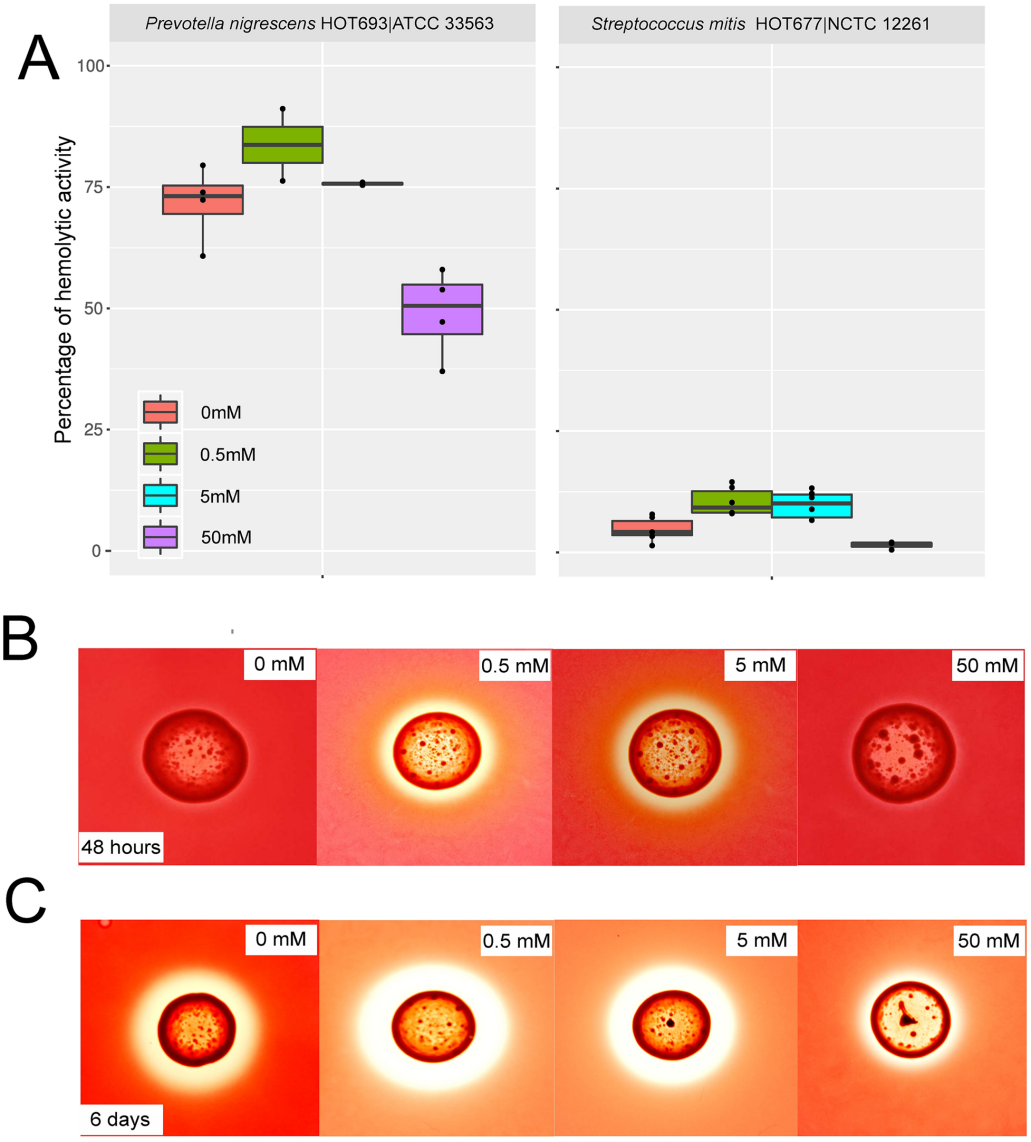
Effect of potassium concentration on hemolytic activity of different bacterial strains. A) Hemolytic activity of supernatants from *P*. *nigrescens* ATCC 33563 and *S*. *mitis* NCTC 1226, as the percentage of lysis of horse erythrocytes with respect to a positive control (100% of activity). Results are from 4 biological replicates for each concentration. B) Hemolytic activity on agar plates with different concentrations of K^+^ added of *P*. *nigrescens* ATCC 33563 after 48 hours of incubation. C) Hemolytic activity on agar plates with different concentrations of K^+^ added of *P*. *nigrescens* ATCC 33563 after 6 days of incubation.

We observed an up-regulation of hemolysins in 45 different species, with the majority belonging to genera *Prevotella* and *Streptococcus* (S4 Table). Among the species presenting high up-regulation of hemolysins were *Prevotella nigrescens* and *Streptococcus mitis*. Increased numbers of *P*. *nigrescens* have been associated with severity of periodontitis [22] and in previous work we showed that *S*. *mitis* expresses a high number of putative virulence factors in periodontitis [10,11]. To determine whether these genera increase hemolysin expression in response to potassium, we assayed hemolytic activity in culture supernatants of *P*. *nigrescens* and *S*. *mitis*. We observed an increase in hemolytic activity in the supernatant of *P*. *nigrescens* and *S*. *mitis* growing in liquid media at 0.5mM and 5mM K^+^ added but an inhibitory effect at 50mM of K^+^ added to the media (Fig 4a and S4 Table). *S*. *mitis* had a lower hemolytic activity. *S*. *mitis* hemolysins tend to accumulate in the cytoplasm rather than in the extracellular environment [23]. These changes in hemolytic activity were not associated with different levels of growth. The final OD_600_ and CFU/mL of the different tubes used for analysis were not significantly different (S5 Table) indicating that activity was increased without a change in the total number of cells.

*P*. *nigrescens* expresses β-hemolytic activity when grown on blood agar with a peak of hemolytic activity on the fifth day of incubation [24]. After 6 days of incubation we observed β-hemolytic activity at all concentrations of K^+^ but with lower activity at 50mM K^+^ in *P*. *nigrescens* (Fig 4b and 4c). Interestingly, as described above, the range where the effect occurs is well defined and once a certain concentration threshold is exceeded the effect is repressed.

### Inflammatory cytokine response of gingival tissue to potassium and dental plaque

To test the effect of potassium on the host plaque interaction, we challenged a three-dimensional gingival multi-layered tissue model with cornified apical layers similar to *in vivo* gingival tissue (EpiGingival GIN-100, MatTek Corp.) with dental plaque. These three-dimensional tissue models have been used in a wide variety of studies and organs [25]. Tissue-engineered 3D culture systems of the oral mucosa provide an organizational complexity that lies between the culture of single cell types and organ cultures *in vivo*. We first confirmed that the histological morphology of the tissue used in the experiments was not altered by the different concentrations of K^+^ or the addition of bacteria. Haematoxylin-eosin stained sections showed a normal morphology of the gingival tissue models in both unchallenged and challenged tissues (S2 Fig). In the tissues that were challenged with dental plaque we observed that bacterial invasion was already occurring regardless of the addition of K^+^ (S3 Fig).

**Fig 5:**
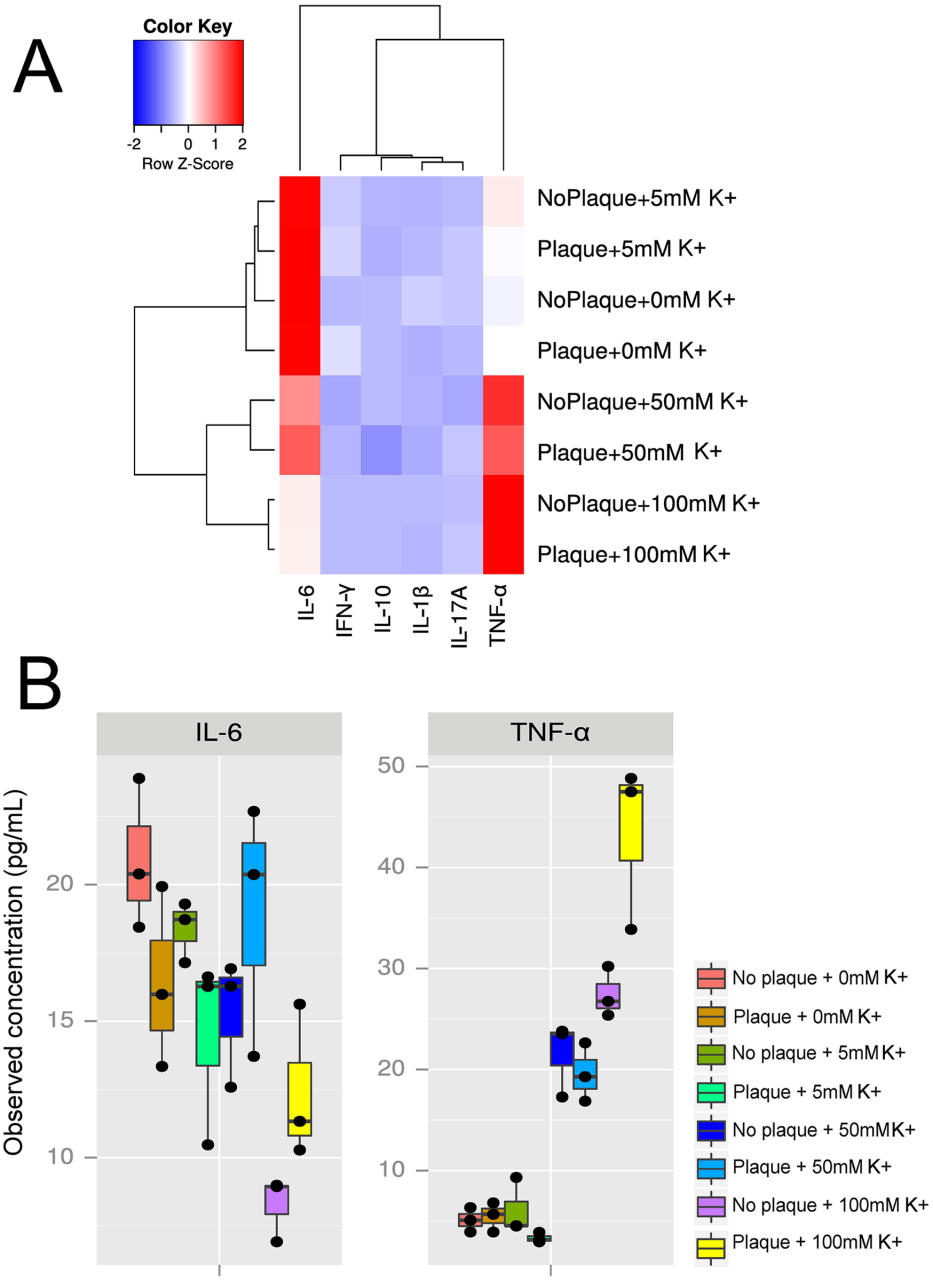
Effect of potassium concentration on gingival cytokine expression. A three-dimensional multilayered gingival tissue model with cornified apical layers (EpiGingival, MatTek Corporation) was used to assess the effect of different concentrations of K^+^ and bacteria on the profiles of expression of different cytokines. A) Heatmap of cytokine expression measured by Luminex under different K^+^ concentrations and presence or absence of bacteria from dental plaque. B) Box plot showing the values of observed concentrations in the media of the different cytokines assayed.

Cytokines are important markers of inflammation. To test the effects of potassium on the inflammatory response to plaque, we measured cytokine production in the presence and absence of plaque and increasing concentrations of potassium. We observed that K^+^ had a major effect on the expression profiles of the cytokines assessed. As shown in Fig 5a, the profiles of expression clustered as a function of K^+^ concentration, regardless of the presence or absence of dental plaque interacting with the tissue. Two cytokines, IL-6 and TNF-α, presented significant differences in their levels of expression associated with the levels of K^+^ (S6 and S7 Tables). IL-6 showed higher levels of expression at 0mM, 5mM and 50mM of K^+^ than at 100mM of K^+^ while TNF-α was significantly up-regulated at 50mM and 100mM of K^+^ added (Fig 5). Most of the other cytokines analyzed (IFN-γ, IL-17A, IL-1β and IL-10) did not change their pattern of expression significantly either with different K^+^ concentrations or with the addition of bacteria to the system (S4 Fig). One of the cytokines, the anti-inflammatory IL-4, was not detected under any of the conditions studied.

Interestingly, interaction analysis of plaque and K^+^ concentration showed that they indeed had an interacting effect on the values of TNF-α and IL-6 but not in the rest of cytokines values (S5 Fig). In case they did not interact we would expect a parallel plot for the lines as it was observed for rest of cytokines. To confirm that conclusion we fit a two-way ANOVA with an interaction term (see S1 Bioinformatic Analisys in Supplementary Information). Those results show evidence of significant interaction between plaque and potassium concentration in TNF-α and IL-6 response (S9 Table).

### Potassium decreases the levels of expression of human beta defensin 3 (hBD-3)

Human β-defensin-3 (hBD-3) is widely expressed in the oral cavity and exerts strong antibacterial and immunomodulatory activities [26]. hBD-3 plays an important role in periodontitis [27] and it is reduced in individuals with severe disease [28]. More importantly, the appropriate expression of hBD-3 peptide may contribute to the maintenance of periodontal homeostasis, possibly through its antimicrobial effect and promotion of adaptive immune responses [29].

**Fig 6:**
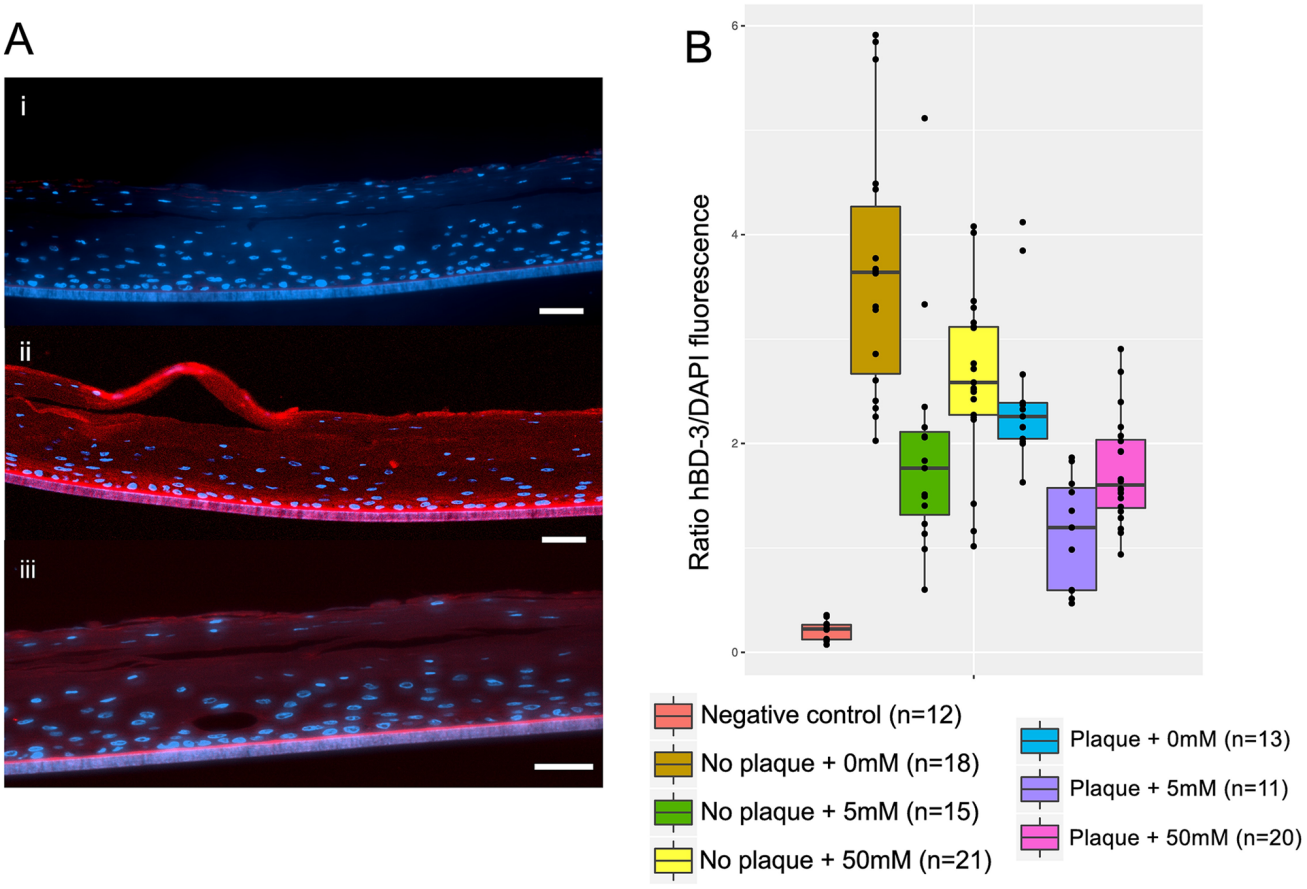
Effect of potassium concentration on expression of human β-defensin 3 (hBD-3) in gingival tissue. A) Immunohistochemistry examples of hBD-3 expression in the three-dimensional (3D) gingival tissue model used for the experiments. i) Negative control, using a non-immunized primary antibody, 0mM K^+^ added and no plaque, ii) 0mM K^+^ added in the absence of plaque, which gave the highest levels of hBD-3 expression, iii) 5mM K^+^ added in the presence of plaque, which gave the lowest levels of hBD-3 expression. B) Box plot showing results of the ratio of hBD-3/DAPI fluorescence measured by immunohistochemistry. The ratio represents a normalized value of hBD-3 expression (see Methods); n: number of different histological sections analyzed. Scale bars = 25 μm.

The three-dimensional tissue model used in these experiments expresses hBD-3 in all layers except the stratum corneum, expresses hBD-1 weakly only in the apical layers, and does not express hBD-2 at all. Using immunohistochemistry we assessed the effect that K^+^ and bacterial plaque had on the levels of expression of hBD-3. Fig 6a shows representative examples of the results. Expression of hBD-3 was observed in all layers including the apical areas of the tissue but was more intense on the basal layers (Fig 6aii and 6aiii). K^+^ had a major effect on hBD-3 expression. The intensity of the signal was normalized by the signal obtained by DAPI, which represents an estimate of the number of cells. The addition of K^+^ by itself inhibited the production of hBD-3 regardless of the presence of plaque. Individually, plaque and potassium each reduced hBD-3 production to similar levels. Combined, plaque and potassium had an additive effect, significantly reducing hBD-3 production more than either treatment alone. (Fig 6b). We compared the statistical significance of those differences using a non-parametric analysis (Kruskall-Wallis correcting for multiple comparisons) and found that all values shown in Fig 6 were significantly different, except for the results with no plaque plus 50mM K^+^ and plaque without K^+^ added, and plaque plus 50mM of K^+^ and no plaque plus 5mM of K^+^ (S8 Table). These results indicate that K^+^ exerts an inhibitory effect on the production of hBD-3, which would clearly weaken the antimicrobial response of the gingival tissue in response to bacterial challenge.

ANOVA analysis revealed that there was not an interaction effect between plaque and potassium on hBD-3 production by the gingival epithelial (F = 0.4148, p = 0.661) (S10 Table, S6 Fig). The test for the effect of the presence of plaque shows a significant effect on the levels of hBD-3 (F = 31.3052, p<0.0001). Similarly, the test for the effect of potassium concentration (F = 23.1869, p<0.0001) indicates a significant effect on the levels of hBD-3.

## Discussion

Dysbiosis of the oral community is mediated by changes in composition and functional activities of the microbiome. In this paper, we show that K^+^ could be an important environmental signal in dysbiosis of the oral microbiome. We have shown that K^+^ induces an increase in pathogenicity of the oral microbiome, and at the same time inhibits mechanisms of defense such as production of hBD-3 that could lead to microbial immune subversion. These results represent a first step in unveiling the role that K^+^ could have in disease.

We are just beginning to understand the mechanisms of oral dysbiosis that lead to disease, where certain organisms can behave as 'keystone-pathogens' (e.g. *P*. *gingivalis*) orchestrating the activities of the rest of the biofilm to their advantage and leading to an aggressive bacterial attack on the gingival tissue [9]. However, the presence of these 'keystone-pathogens' does not necessarily explain why disease is initiated or progresses, since they can be detected in healthy individuals and non-progressing sites [30,31]. In addition, other environmental signals may be responsible for the initiation of the shift from commensal to dysbiotic communities. Our findings provide a first report of one of such key signals leading to dysbiosis. Despite the novelty of the current study, some limitations must be considered when interpreting the results. In our metatranscriptome and tissue-engineered 3D culture systems experiments we used plaque from only one human volunteer and other plaque samples may behave differently and we performed short term experiments that resemble an acute response rather that the chronic nature of periodontitis. In future studies it would be more appropriate to perform long-term steady-state experiments. However, to this date it is extremely difficult to maintain reproducible oral microbial communities that resemble the complexity of the oral microbiome for long periods of time [32–34]. There are models for oral microbial communities but the selection of the organisms used on those models is mainly based on how easy is to grow them than in the real importance of those organisms for the homeostasis of the microbial community. Moreover, even the addition of few new members would completely alter the expression profiles of the rest of members of the oral biofilm [35].

Using metatranscriptome analysis of the oral microbiome during periodontitis progression, we identified several metabolic signatures associated with periodontitis progression [11] and with severe periodontitis [10], being potassium ion transport one of the most significant in our analysis of GO biological processes. Although we can not discard the possibility that other ions could, and likely do have an effect on virulence, the fact that we only observed changes in expression of potassium ion transport in our *in vivo* studies at the time of disease progression and severe periodontitis seems to indicate that this particular ion acts as an important signal in the transition from health to dysbiosis [10,11]. Whether potassium exerts its effect directly or through an increase on osmolarity K^+^ has to be revealed in future studies.

Potassium, which plays an essential role in cellular homeostasis, is the most abundant ion in the cytoplasm of prokaryotic and eukaryotic cells. The internal concentration of K^+^ in a typical mammalian cell is 139mM [36]. Maintenance of high internal K^+^ concentration and consequently potassium uptake are of crucial physiological significance. Moreover, K^+^ acts as a cytoplasmic signaling molecule, activating and/or inducing enzymes and transport systems. The signal could be ionic strength or specifically K^+^ itself [37]. However, not until recently it has been shown that ion potassium channels in bacteria enable bacterial communication in biofilms and that potassium is the key signal in propagating the signal through a monospecific biofilm of *Bacillus subtilis* [38]. The natural environment in which the periodontal biofilm grows, the subgingival crevice, is in direct contact with the gingival crevicular fluid (GCF) rich in inflammatory mediators that are important in maintaining the homeostasis of the system. Levels of K^+^ in the GCF increase with severity of periodontal disease while levels of other ions such calcium remain stable [12]. In GCF from healthy patients, Kaslick et al. reported mean values of K^+^ of 10mM [13], while in severe periodontitis K^+^ concentrations of more than 20mM have been reported [13,14]. Although the source of such levels of K^+^ in GCF is not known it may come from host cells lysis, which could be sensed by the oral microbiome as a signal of tissue damage that in turn triggers up-regulation of genes involved in pathogenicity and induction of a local immune response, thus initiating a positive feedback loop in which lysis of host cells by microbial proteolytic activity or tissue immune responses lead to the release of more K^+^ into the GCF. Tissue irritation, caused for instance by the placement of a ligature in murine animal models, leads to disruption of the normal commensal microbiota [39]. Plaque accumulation on the ligature has been suggested as the primary reason for the initiation of periodontitis in those models, nonetheless release of K^+^ may also a role in inducing virulence in the oral microbiome.

In the present study we showed that an increase in K^+^ in the *ex vivo* model used to mimic the oral environment lead to a rapid metabolic response of the microbial community as a whole, increasing the activity of known putative periodontal pathogens. Using metatranscriptomic analysis of biofilms at high concentrations of potassium we show an up-regulation of community-wide virulence factors expression such as iron transport and motility. Virulence of pathogenic organisms is related to the availability of iron, therefore, microbial iron acquisition mechanisms are an important determinant of infection potential. In the major periodontopathogen *Porphyromonas gingivalis* iron limitation up-regulates the genes involved in iron uptake and its ability to invade host cells [40,41]. It is also well established that flagellated bacteria are abundant in samples from patients having periodontal disease [42]. Motility is not considered to be a classic virulence factor of bacteria. However, in other major periodontopathogen, *Treponema denticola*, motility is a key element for the virulence of the bacterium during disease progression [43,44]. Additionally, we have recently found that cobalamin biosynthesis and oligopeptide transport activities are associated with the progression of periodontitis [11].

The increase in virulence was experimentally confirmed by the increase in hemolytic activity of specific bacteria that had previously showed up-regulation of hemolysins in periodontitis progression [11] (*P*. *nigrescens* and *S*. *mitis*) as well as the whole oral biofilm. Interestingly, a high concentrations of potassium (50 mM K^+^) the isolated species *P*. *nigrecens* and *S*. *mitis* had the opposite behavior that the whole dental plaque. While hemolytic activity was inhibited in *P*. *nigrescens* and *S*. *mitis* at 50 mM K^+^ it was induced in the whole dental plaque biofilm sample, most likely due to the different behavior that bacteria show in a mixed biofilm and isolated from it [45].

Proteolysis was one of the community-wide activities observed as up-regulated in the presence of high concentration of K^+^, which has been shown to contribute to virulence, and most importantly in degradation of host extracellular matrix proteins that leads to a loss of the epithelial barrier in the oral cavity [46,47]. Our results confirm previous observations where dysbiotic activities were mainly driven by organisms that are generally considered commensals [10,11]. Thus in our previous study of periodontitis progression we observed that the vast majority of up-regulated putative virulence factors were expressed by organisms not consider periodontal pathogens [11]. Indeed, adding K^+^ increased the up-regulation of hemolysins and hemolytic activity in *S*. *mitis*, which under the right environmental conditions is capable of causing severe infectious diseases outside the oral cavity [48,49].

The short-term response, akin to an acute response, to the presence of K^+^ of a multilayered tissue model similar to *in vivo* gingival tissue was similarly rapid. We demonstrated that high levels of potassium modulated the response of a gingival epithelial three-dimensional (3D) model to the bacterial challenge altering the expression of cytokines. Oral keratinocytes express a variety of pro-inflammatory cytokines, including IL1-α, IL-1β, IL-6, IL-8 and TNF-α [50] after just a few hours of incubation. Among the host mediators produced after microbial recognition, innate immunity cytokines such as TNF-α, IL-1, and IL-6 were the first to have their roles in periodontal disease pathogenesis unraveled [51]. Our results showed a pro-inflammatory profile of cytokine expression associated with elevated concentrations of K^+^, while the anti-inflammatory cytokines IL-10 and IL-4 were not altered by the presence either K^+^ or bacteria. In fact IL-4 was not detected under any of the conditions assayed. IL-4 has been reported to possess a protective role in periodontal disease [52] and could mediate the remission or improvement of periodontal lesions [53]. The literature on the protective role for IL-10 is much stronger than for IL-4. Knockouts of IL-10 have rapid periodontal destruction in response to microbial challenge while knockouts of IL-4 do not produce the same effect [54] and have naturally-occurring IBD [55].

On the other hand, the profiles of expression of two assessed cytokines: TNF-α and IL-6, were altered by the concentration of K^+^ in the medium. TNF-α and IL-6 are major pro-inflammatory cytokines, although it has been suggested that IL-6, under certain conditions, could act also as anti-inflammatory [50,56]. Expression of TNF-α was up-regulated at high concentrations of K^+^ but not altered by the presence of bacteria, indicating that K^+^ by itself had a pro-inflammatory effect on the gingival tissue model. Data from human studies as well as from animal models clearly demonstrated that TNF-α plays a central role in inflammatory responses and the loss of connective tissue attachment [57]. TNF-α is present at high levels in both gingival crevicular fluid (GCF) and tissues in periodontitis, where positively correlates with matrix metalloproteinases and receptor activator of nuclear factor kappa-B ligand expression [57,58]. Moreover, TNF-α plays a key role on bone resorption by promoting osteoclastogenesis by stimulating RANKL expression and bone-resorbing osteoclasts exposed to permissive levels of RANKL [59–61].

IL-6 also showed significant changes under different K^+^ concentrations. Although IL-6 and TNF-alpha are normally thought of as pro-inflammatory cytokines, IL-6 followed a complete different pattern of expression than TNF-α, being expressed at higher levels at low concentrations of K^+^ This results seem to indicate that in our gingival epithelial model TNF-α and IL-6 are independently regulated. Previous studies have shown that in some instances IL-6 and TNF-alpha are coordinately regulated, while in other instances they can be regulated independently. Increased levels of IL-6 have been detected in the crevicular fluid of active sites compared with healthy sites of patients with refractory periodontitis [62] and exposure to lipopolysaccharide from the *P*. *gingivalis* induces elevated levels of IL-6 and TNF-α in primary gingival mouse cell lines [63]. Nonetheless, it has also been shown that IL-6 inhibits production of TNF-α in culture human monocytes, as a part of a TNF-α/IL-6 regulatory circuit [64].

Our findings showed that the presence of bacteria increased IL-6 expression regardless of K^+^ concentration. Expression of IL-6 is induced in epithelial cells following adhesion by organisms as diverse as *E*. *coli* and *H*. *pylori* [65]. More interesting is the fact that IL-6 can also be induced by bacterial invasion [66]. We observed invasion in all K^+^ assayed which could explain the induction of IL-6 when dental plaque was added to the tissue.

A central aspect of both homeostasis and dysbiosis in the oral cavity is the subversion of the immune response by the oral microbiome to overcome the host mechanisms of defense. Among them we find immunosuppression, complement subversion and blocking the production of human β‐defensins [67].

hBD-3 is a potent antibacterial peptide that is expressed at similar levels in samples of both healthy and inflamed gingival tissue [26]. We observed a decrease in hBD-3 expression when K^+^ was added as well as additional inhibition when dental plaque was present. It has been shown that the oral pathogen *T*. *denticola* suppresses the expression of hBD-3 in gingival epithelial cells but has no effect on the production of TNF-α, adding to the virulence of this bacterium and its role during the progression of periodontal inflammation [68]. In the presence of LPS, hBD-3 (but not hBD2), effectively inhibit TNF-α and IL-6 accumulation [69]. The suppression of hBD-3 by K^+^ and bacteria my favor biofilm growth and proliferation during periodontitis.

Despite the limitations of our study, these findings represent an important advance in our understanding of signaling during the initial stages of breakdown of host-microbiome homeostasis in the oral cavity, and may also be important in other polymicrobial inflammatory diseases such as IBDs where microbial dysbiosis is an essential factor in the evolution of pathology.

### Conclusions

Our findings highlight the importance of ion potassium as a signal for dysbiosis in periodontitis. In the presence of high concentration of ion potassium we observed an increase in virulence of the whole microbial community, which agrees with our previous *in vivo* observations on severe periodontitis and progression of the disease. Moreover, we demonstrated the effect of potassium on the expression of virulence factors of isolated oral microorganisms including *S*. *mitis*, which is considered a commensal under normal conditions. Furthermore, pro-inflammatory cytokines were up-regulated as a response to the presence of K^+^ and bacteria and expression of the antimicrobial peptide hBD-3 was inhibited by both potassium and dental plaque. Future studies are needed to confirm our results on a periodontitis mouse model as well as to identify the mechanisms by which the oral biofilm senses the different levels of potassium in the environment

## Materials and methods

### Challenging the oral microbiome with ion potassium

To assess the effect that K^+^ has on the oral microbiome we performed metatranscriptome analysis of its effect on dental plaque. Dental plaque from a periodontally healthy subject sample was resuspended in his own saliva, vortexed for 30 seconds and split in 3mL aliquots, each placed in a well of a 6 well, flat bottom Corning Costar cell culture plate. To 3 of the well containing the saliva/plaque suspension we added KCl to a final K^+^ concentration of 50mM and the other 3 well containing saliva/plaque suspension were used as controls. The culture plate containing the samples was incubated at 37°C for 3 hours under anaerobic conditions. Cells were collected by centrifugation at 10,000 x g for 5 minutes and RNA was extracted immediately for further analysis.

### Next generation sequencing (NGS)

Detailed protocols for community RNA extraction, RNA amplification and Illumina Sequencing are described in Yost et al. [11].

### Selection of genomes in databases

Genomes of archaea and bacteria as well as their associated information were downloaded from the HOMD database server (http://www.homd.org/), the PATRIC ftp server (https://www.patricbrc.org/) [70] and the J. Craig Venter Institute (www.jcvi.org). A total of 524 genomes from 312 species of bacteria and 2 genomes from 1 archaea species were used in the analysis. Detailed explanation of genomes is reported in Yost et. al. [11].

### Short reads sequence alignment analysis

Low-quality sequences were removed from the query files. Fast clipper and fastq quality filter from the Fastx-toolkit (http://hannonlab.cshl.edu/fastx_toolkit/) were used to remove short sequences with quality score >20 in >80% of the sequence. Cleaned files were then aligned against the bacterial/archaeal database using bowtie2. We generated a.gff file to map hits to different regions in the genomes of our database. Read counts from the SAM files were obtained using bedtools multicov from bedtools [71].

### Phylogenetic analysis of the metatranscriptome

Counts from the mRNA libraries were used to determine their phylogenetic composition. Phylogenetic profiles of the metatranscriptomes were obtained using the latest version of Kraken [17]. Phylogenetic profiles were used to identify significant differences between active communities under the different conditions studied. We performed linear discriminant analysis (LDA) effect size (LEfSe) as proposed by Segata et al. [18] with default settings except that LDA threshold was raised to 3 to increase the stringency of the analysis.

### Differential expression and gene ontology (GO) enrichment analysis

To identify differentially expressed (DE) genes from the RNA libraries, we applied non-parametric tests to the normalized counts using NOISeqBio function of the R package 'NOISeq' with 'tmm' normalization, with batch and length correction and removing genes whose sum of hits across samples was lower than 10. We used a threshold value for significance of q = 0.95, which is equivalent to a FDR adjusted p-value of 0.05 [72].

To evaluate functional activities differentially represented we mapped the DE genes to Gene Ontology (GO) terms (http://www.geneontology.org/). GO terms for the different ORFs were obtained from the PATRIC database (https://www.patricbrc.org/). GO terms not present in the PATRIC database and whose annotation was obtained from the HOMD database or from the J. Craig Venter Institute were acquired using the program blast2GO under the default settings [73]. Enrichment analysis on these sets was performed using the R package 'GOseq', which accounts for biases due to over-detection of long and highly expressed transcripts [73]. Gene sets with ≤ 10 genes were excluded from analysis. We used the REVIGO web page [74] to summarize and remove redundant GO terms. Only GO terms with FDR adjusted p-value < 0.05 in the 'GOseq' analysis were used.

### Three-dimensional (3D) human gingival tissue model

We used the EpiGingival GIN-100 (MatTek Corp.) a multilayered tissue model with the apical layers cornified, similar to *in vivo* gingival tissue. The tissues used in these experiments had more than 10 layers of cell with 300,000 to 500,000 cells per tissue. After arrival, the tissues were maintained overnight in a humidified incubator at 37°C and in the presence of 5% CO2 in Dulbecco's modified Eagle medium (DMEM) supplemented with 10% (vol/vol) fetal bovine serum (GIBCO/BRL) and 1% (vol/vol) penicillin-streptomycin (GIBCO/BRL). Next day the tissues were washed 3 times with PBS to remove any traces of antibiotics and were reinoculated with DMEM without K^+^ (USBiological Life Sciences D9800-15) or antibiotics. We challenged the tissues with different K^+^ concentrations (0, 5, 50 and 100mM) and with bacteria from dental plaque and saliva. 4 different tissues were used for all concentrations. Supra and subgingival plaque from the same healthy volunteer used in all experiments was collected in saliva and diluted in DMEM -K to a McFarland standard #1 value (McFarland Standards Gibson Laboratories), which is equivalent to approximately 3 x 10^8^CFU/mL. Based on the number of cells per tissue supplied by MatTek Corp. (see above) we inoculated with a multiplicity of infection (MOI) of 100, which had been used previously with success with other oral bacteria in invasion experiments [75].

At the end of the experiment the tissues were fixed with 4% formalin for 24 hours, paraffin embedded and cut into 5μm slices that were mounted on poly-lysine glass slides. Some slides were haematoxylin-eosin stained to check the integrity of the tissues. The rest were used for FISH analysis and immunohistochemistry as describe below.

### Quantification of cytokines using multiplexed bead immunoassay (Luminex)

Cytokine levels from 3 biological replicates of the medium surrounding the tissue cultures under the conditions described above were determined using MILLIPLEX MAP Human Cytokine/Chemokine Magnetic Bead Panel-Immunology Multiplex Assay (EMD Millipore, Billerica, MA, USA). Seven cytokines: IFN-γ, IL-10, IL-17A, IL-4, IL-6, IL-1β and TNF-α were measured. Samples were thawed at 4°C prior to assay and kept on ice throughout the assay procedures. Manufacturers’ protocols were followed for all panels, with a general protocol as follows. Reagents were prepared as per kit instructions. Assay plates (96-well) were loaded with assay buffer, standards, samples, and beads and then covered and incubated on plate shaker (500 rpm) overnight at 4°C. After primary incubation, plates were washed twice and then detection antibody cocktail was added to all wells; the plates were covered and left to incubate at room temperature for 1 hour on plate shaker. After one hour incubation, streptavidin-phycoerythrin fluorescent reporter was added to all wells, plates were covered and incubated for 30 minutes at room temperature on plate shaker. Plates were then washed twice and beads were resuspended in wash buffer, placed on shaker for 5 minutes, and then read on Bio-Plex200 following manufacturers’ specifications and using Bio-Plex Manager software v6.0.

### Immunohistochemistry

Mounted slides were deparaffinized using standard protocols. Antigen retrieval was performed in 10mM Na Citrate pH 6 buffer in a microwave with an initial cycle of 2 minutes at 80% power and a final cycle of 8 minutes at 40% power. Slides were blocked on blocking solution (2% goat serum in PBS) for 1 hour, washed three times with PBS and incubated overnight with Anti hBD-3 antibody L3-18b-E1 (Abcam, Cambridge, MA, USA) at 4°C. As a negative control we used as a primary antibody a polyclonal rabbit IgG whose serum was obtained from naive (non-immunized) rabbits (RD Systems, Minneapolis, MN, USA). After incubation with the primary antibody slides were washed three times in PBS and incubated with goat anti-Rabbit IgG (H+L) secondary Antibody, Alexa Fluor 594 conjugate (LifeTechnologies). We finally counter stained the slides with DAPI (100ng/mL) for 10 minutes before analysis. Tissues were dried and mounted with Prolong Gold anti-fade (Life Technologies). Images were captured using an Inverted Widefield Fluorescence—Zeiss Cell Observer Z using a 10x and 20x objectives and analyzed using Fiji Software [76]. The number of histological sections analyzed are indicated in Fig 6 legend. Sections for each condition tested came from a single three-dimensional multilayered gingival tissue model with cornified apical layers (EpiGingival, MatTek Corporation). Because of the low magnification used for analysis we analyzed one field per section to avoid fields with wrinkles or creases on the slide that could interfere with fluorescence measurements. Files with.czi extension were open in Fiji with ‘Autoscale’, ‘Split channels’ and ‘Color mode = colorized’ options marked. Brightness and contrast of the images were auto-adjusted, merged (channels ‘red’ and ‘blue’) and converted to RGB and analyzed using ‘Color histogram’. Results for the color histogram gives values of intensity for the different channels. We used the ratio of red fluorescence (Alexa Fluor 594 from hBD-3 expression) and blue fluorescence (DAPI from the cells' nuclei) as an estimate of the levels of expression of hBD-3 in the tissues.

### Fluorescent in situ hybridization (FISH)

We performed FISH on the mounted slides with a universal probe for bacteria labeled with EUB probe (EUB388 5′-GCT GCC TCC CGT AGG AGT) [77] (Life Technologies). The hybridization oven was pre-warmed to 46°C and humidifying solution (20% formamide non-HiDi in water) was added to the hybridization chamber. Slides were placed in hybridization chamber and covered with 40μl of probe mixture (0.9 M NaCl, 0.02 M Tris pH 7.5, 0.01% SDS, 20% Hi-Di formamide and 2pmol/μl) on top of whole mount. The hybridization chamber was sealed with parafilm and samples were incubated at 46°C for 3 hours. After hybridization in fume hood excess of hybridization solution from the slides was drained and slides were washed in 50 ml of pre-warmed wash buffer (215mM NaCl, 20mM Tris pH 7.5, 5mM EDTA) to 48°C for 15 minutes in the hybridization oven. Finally, slides were dipped in ice-cold water followed by a final dip in 100% ethanol at room temperature. The slides were air-dried and mounted in Prolong Gold antifade (LifeTechnologies) before being observed under the microscope as described above.

### Deep-sequencing analysis of 16S rRNA (HOMINGs)

DNA was extracted from the fresh plaque inoculum used in the plate hemolysis assays as well as from the cultures growing and showing hemolysis after 6 days of incubation as described below. DNA extraction was performed using Ultraclean Microbial DNA Isolation kit. (MoBio, Carlsbad, CA) following manufacturers’ specifications. We used HOMINGS (http://homings.forsyth.org/index2.html) for species-level identification of oral bacteria using 341F (5' ATGATACGGCGACCACCGAGATCTACACTATGGTAATTGTCCTACGGGAGGCAGCAG) and 806R (5' CAAGCAGAAGACGGCATACGAGATNNNNNNNNNNNNAGTCAGTCAGCCGGACTACHVG GTWTCTAAT) primers to amplify the V3-V4 region of the 16s rRNA gene. The underlined stretch of 12N are designated barcode sequences. HOMINGS uses species-specific, 16S rRNA-based oligonucleotide 'probes', designed to target oral species, as a database in a BLAST program ('ProbeSeq' for HOMINGS) to identify the frequency of oral bacterial targets.

### Quantification of bacterial load by real-time PCR (qPCR)

Bacterial load was quantified by qPCR following the method described in Nadkarni et al. [78]. Total DNA was extracted as described above. *Escherichia coli* DNA was used as the standard for determining bacterial number by qPCR. Amplification and detection of DNA by real-time PCR were performed with iCycler (BioRad) using optical grade 96-well plates. Triplicate samples were used for the determination of DNA by qPCR. The PCR reaction was performed in a total volume of 20 μl using the TaqMan Universal PCR and Master Mix (Integrated DNA Technologies), containing 100 nM of each of the universal forward and reverse primers and the fluorogenic probe.

### Hemolysis assays

Two different hemolysis assays were performed to assess the activity of individual organisms and whole dental plaque. The first was a plate hemolysis assay that gave a visual assessment of the hemolytic activity on agar plates to which different amounts of K^+^ were added. The second was a quantitative assay that measures release of hemoglobin from erythrocytes due to hemolysis. Agar hemolysis assays: we used *P*. *nigrescens* ATCC 33563 as a model organism. *P*. *nigrescens* was grown O/N in Schaedler Anaerobic Broth (Oxoid, Thermo Scientific, Lenexa, KS) and 10μl of the culture were spotted on horse blood TSBY plates with 0, 0.5, 5 and 50 mM of K^+^ added. The plates were incubated at 37°C under anaerobic conditions and checked at 48 hours and 6 days. For the biofilm assay we used fresh collected dental plaque from the same healthy individual who was used for the rest of experiments and resuspended in 100μl Schaedler Broth (Oxoid, Thermo Scientific, Lenexa, KS). 10μl of the suspension were spotted on horse blood TSBY plates with 0, 0.5, 5 and 50 mM of K^+^ added. The plates were incubated at 37°C under anaerobic conditions and checked at 48 hours and 6 days.

**Quantitative hemolysis assays:** To quantitatively measure hemolytic activity we used supernatants from *P*. *nigrescens* ATCC 33563 and *S*. *mitis* NCTC 1226. We followed the protocol describe by Maltz and Graf [79] with minor modifications. Briefly, Schaedler Anaerobic Broth (Oxoid, Thermo Scientific, Lenexa, KS) diluted to 50% with water was pre-reduced in an anaerobic chamber for 48 h. Prior to pre-reduction the different concentrations of K^+^ were added (0, 0.5, 5 and 50mM K^+^). *P*. *nigrescens* cells were inoculated from TSBY plates to OD_600_ of 0.2 and grown O/N 37°C under anaerobic conditions. *S*. *mitis* was grown on Todd-Hewitt Yeast Broth (THY: Bacto Todd–Hewitt Broth supplemented with 0.2% yeast extract) diluted to 50% with a minimal streptococci medium [80]. THY/MM media was pre-reduced in anaerobic chamber for 48 hrs. As mentioned above, prior to pre-reduction the different concentrations of K^+^ were added (0, 0.5, 5 and 50mM K^+^). *S*. *mitis* cells from TSBY plate were resuspended in 1.0 ml THY/MM and 100μl were added to tubes containing 3.0 ml of the pre-reduced THY/MM to OD_600_ of 0.2 and grown O/N at 37°C under anaerobic conditions.

Horse red blood cells (Horse RBC; Northeast Laboratory Services) were washed 3 times in 1.0 ml PBS and resuspended in PBS to final concentration 10% v/v. The O/N cultures were spun (5 min, 7,500 rpm) and 250 μl of culture supernatant was added to 250 μl of washed erythrocytes and incubated for 3h at 37°C. Horse RBC were also incubated with 250 μl PBS (negative control) or ultra pure dH_2_O (positive control). Reactions aliquots were centrifuged and 100 μl of each biological replicate were analyzed for hemolytic activity at OD_540_ with a BioTek Synergy HT plate reader.

### Statistical analysis

We used the R package 'agricolae' to perform the non-parametric multiple comparison Kruskal-Wallis analysis on our results. Shapiro test analysis of our results showed that they did not follow a normal distribution. FDR adjusted p-value were obtained by setting the 'p.adj' argument of the 'kruskal' function as “fdr”. A cut-off value of 0.05 was used to determine the significance of the results. We used a Two-way ANOVA interaction test in R to assess whether plaque and potassium had an additive or an interaction effect on levels of cytokines and hBD-3. The detailed protocol is described in the section ‘Two-way ANOVA interaction test in R’ in the S1 Bioinformatic Analysis file of the supplementary information.

## Supporting information

S1 Bioinformatic analysis Detailed description of the complete bioinformatic analysis performed in the present study. (PDF)

S1 Fig Sequencing depth and expression quantification. NOISeq qualitity analysis of the sequencing libraries. A) Saturation plot for protein-coding genes for all samples. B) Sensitivity plot. Percentage of features having more than 0, 1, 2, 5 and 10 counts per million (CPM).
(PDF)

S2 Fig Haematoxylin-eosin stained sections of the 3D gingival tissue model. A) 0mM No plaque 20x B) 50mM K^+^ No Plaque 20x C) 0mM K^+^ Plaque 20x D) 50mM K^+^ Plaque 20x. Key: SC, Stratum corneum, BC, Basal cells, MM, Microporous membrane.
(PDF)

S3 Fig FISH of tissue culture invasion. Brightly fluorescent cell-associated bacteria seen with the EUB338 probe. Green autofluorescence background from the tissue. A) 0 K^+^ added. B) 50mM K^+^ added. Magnification, ×400.
(PDF)

S4 Fig Effect of potassium concentration on gingival cytokine expression. A three-dimensional multilayered gingival tissue model with cornified apical layers (EpiGingival, MatTek Corporation) was used to assess the effect of different concentrations of K^+^ and bacteria on the profiles of expression of different cytokines. Cytokine expression was measured by Luminex under different K^+^ concentrations. Box plots show the values of observed concentrations in the media of the different cytokines assayed.
(PDF)

S5 Fig Interaction analysis Labels (plaque/no plaque) and potassium concentration. A) Interaction plots displaying the levels of one factor on the x-axis and the mean response on the y-axis for all cytokines analyzed. B) Interaction plots displaying the levels of one factor on the x-axis and the mean response on the y-axis for IL-6. C) Interaction plots displaying the levels of one factor on the x-axis and the mean response on the y-axis for TNF-α.
(PDF)

S6 Fig Interaction analysis plaque/no plaque and potassium concentration. A) Boxplots showing values based on label (plaque/no plaque) and potassium concentrations. B) Interaction plots displaying the levels of one factor on the x-axis and the mean response on the y-axis.
(PDF)

S7 Fig Immunohistochemistry of hBD-3 expression in the three-dimensional (3D) gingival tissue model used for the experiments. Representative images of all treatments performed. We used the red fluorescence for hBD-3 expression (Alexa Fluor 594) and blue fluorescence (DAPI) to stain cells' nuclei. Scale bars = 25 μm.
(PDF)

S1 Table Summary of molecular function gene ontolology (GO) terms enriched in the presence of potassium. 50mM of K^+^ were added to dental plaque on saliva and incubated for 3 hours at 37°C in anaerobic conditions. GO terms enrichment analysis was performed as described in the Methods section.
(PDF)

S2 Table Up and down-regulated putative virulence factors after the addition of K^+^ (50mM K^+^) to dental plaque incubated in saliva. NOISeqBio Results of all differentially expressed genes.
(XLSX)

S3 Table Hemolytic activity analysis. Kruskal-Wallis analysis corrected for multiple comparisons of percentage of hemolytic actitivy and growth measured as OD_600_ and CFUs after 12 hours of incubation. Tables show corrected p-values. In yellow are comparisons that were statistically significant with a p-value < 0.05.
(PDF)

S4 Table Metagenomic composition of the different hemolytic communities based on HOMINGs analysis. Number represent % of the community.
(XLSX)

S5 Table A) Total number of bacteria measured by qPCR in inocula and agar plates used on hemolytic activity of dental plaque experiments. B) p-values of Kruskal-Wallis analysis corrected for multiple comparisons. In yellow are differences that were statistically significant.
(PDF)

S6 Table Differences in cytokine expression due to K^+^ concentration and presence or absence of dental plaque. Kruskal-Wallis analysis corrected for multiple comparisons. In yellow are comparisons that were statistically significant with corrected p-value < 0.05. In green are comparisons that were statistically significant with corrected p-value < 0.1. NP, no plaque. P, plaque.
(PDF)

S7 Table A) IL-6 and TNF-α expression differences due to the addition of dental plaque. Kruskal-Wallis analysis corrected for multiple comparisons. In yellow are comparisons that were statistically significant with corrected p-value < 0.05. B) IL-6 and TNF-α expression differences due to K^+^ concentration regardless of the addition of dental plaque. Kruskal-Wallis analysis corrected for multiple comparisons. In yellow are comparisons that were statistically significant with corrected p-value < 0.05. C) IL-6 and TNF-α expression differences at high (50-100mM) and low (0-5mM) K^+^ concentration regardless of the addition of dental plaque. Kruskal-Wallis analysis corrected for multiple comparisons. In yellow are comparisons that were statistically significant with corrected p-value < 0.05.
(PDF)

S8 Table Kruskal-Wallis analysis corrected for multiple comparisons for expression levels of hBD-3. See Fig 6. In yellow are comparisons of the effects of the different concentrations of K^+^ on hBD-3 expresssion that were statistically significant.
(PDF)

S9 Table Analysis of variance tables. Output of the two-way ANOVA analysis to assess for significant interactions in expression levels of IL-6 and TNF-α. See S6 Fig. In yellow are comparisons that were statistically significant.
(PDF)

S10 Table Analysis of variance table. Output of the two-way ANOVA analysis to assess for significant interactions in expression levels of hBD-3. See Fig 6. In yellow are comparisons that were statistically significant.
(PDF)
