# Supplementary material for: Potassium is a key signal in host-microbiome dysbiosis in periodontitis
Source: PLoS Pathog. 2017 Jun 20;13(6):e1006457. doi: 10.1371/journal.ppat.1006457 (PMC5493431; doi:10.1371/journal.ppat.1006457)
Supplement: S1 Bioinformatic analysis — (PDF) [file ppat.1006457.s001.pdf]

```
#######################################################################################
########################### BIOINFORMATIC ANALYSIS ###################################
#######################################################################################

#######################################################################################
######################### Removal of low-quality sequences #################################
#######################################################################################

$ fastx_clipper -i Sample_1_R1.fastq -o Sample_1_R1_clipped.fastq -l 50 -Q33 -v -a
AAAAAAAAAAAAAAAAAAAAAAAAAAAAAAAAAAAA

$ fastq_quality_filter -i Sample_1_R1_clipped.fastq -o Sample_1_R1_clean.fastq -q 20 -p 80 -Q 33 -v

####### Combine the clean pair-end sequences ######

$ python fastqcombinepairedend.py "@M01" " " Sample_1_R1_clean.fastq Sample_1_R2_clean.fastq

#######################################################################################
##############################  bowtie2 aligment #########################################
#######################################################################################

$ bowtie2 --local -N 1 -L 20 -D 30 -t -R 3 -i S,1,0.25 --no-unal -p40 -x ./oral_microbiome_genomes -1
Sample_1_R1_clean_trimmed_clipped_stillpaired.fastq -2
Sample_1_R1_clean_trimmed_clipped_stillpaired.fastq -S Sample_1.sam

$ samtools view -@20 -bS Sample_1.sam > Sample_1.bam

$ samtools sort -@35 Sample_1.bam Sample_1Sorted

$ samtools index Sample_1Sorted.bam

$ bedtools multicov -bams Sample_1Sorted.bam .... Sample_NSorted.bam -bed oral_microbiome_CDS.gff >
gene_counts.gff ##### Generates the counts table

$ sed 's/^.*locus_tag=//' gene_counts.gff > gene_counts.tab

#################### Number of mapped reads #############################################

$ samtools view -F 0x4 Sample_1Sorted.bam | cut -f1 | sort | uniq | wc -l >> Number_of_mapped_reads.txt


#######################################################################################
#################### NOISeqBio in R Exploratory analysis ####################################
#######################################################################################

> library(NOISeq)

> mycounts<-read.table('gene_counts.tab', header=TRUE, row.names=1)

> mycounts=mycounts[which(rowSums(mycounts) > 0),] # Remove 0S

> mylength<-read.table('gene_length_NOISeq.txt', header=FALSE) ######## In case you use RPKM or TMM
length correction
```

```
> myfactors = data.frame(condition = c("CB","CB","CB","PB","PB","PB","PB"), replicate =
c("CB1","CB2","CB3","PB1","PB2","PB3","PB4"))

mylength<-read.table('gene_length_NOISeq.txt',header=FALSE, row.names=1)

mydata <- readData(data = mycounts,factors = myfactors, length = mylength)

mycountsbio = dat(mydata, factor = NULL, norm=FALSE, type = "countsbio")

mysaturation = dat(mydata, k = 0, ndepth = 7, type = "saturation")

QCreport(mydata, samples = NULL, factor = "condition") # This final line produces a quality control report

##################### Repeat with normalized data to see which one works best #################

#######################################################
## RPKM normalization
#######################################################

mylength<-t(mylength)

myrpkm = rpkm(mycounts, long = mylength, lc = 1, k = 0)

mydata <- readData(data = myrpkm,factors = myfactors)

mycountsbio = dat(mydata, factor = NULL, norm=TRUE, type = "countsbio")

mysaturation = dat(mydata, k = 0, ndepth = 7, type = "saturation")

QCreport(mydata, samples = NULL, factor = "condition")


#######################################################
## TMM normalization with no length correction
#######################################################

mytmm = tmm(mycounts, long = 1000, lc = 0, k = 0)

mydata <- readData(data = mytmm,factors = myfactors)

mycountsbio = dat(mydata, factor = NULL, norm=TRUE, type = "countsbio")

mysaturation = dat(mydata, k = 0, ndepth = 7, type = "saturation")

QCreport(mydata, samples = NULL, factor = "condition")
```

```
##################################################
## TMM normalization with length correction
##################################################

mylength<-t(mylength)

mytmm = tmm(mycounts, long = mylength, lc = 1, k = 0)

mydata <- readData(data = mytmm,factors = myfactors)

mycountsbio = dat(mydata, factor = NULL, norm=TRUE, type = "countsbio")

mysaturation = dat(mydata, k = 0, ndepth = 7, type = "saturation")

QCreport(mydata, samples = NULL, factor = "condition")

###############################################################################
#################### Differential expression analysis with NOISeqBio in R ###################
###############################################################################

> library(NOISeq)

> mycounts<-read.table('gene_counts_CB_vs_PB.tab', header=TRUE, row.names=1)

> mycounts=mycounts[which(rowSums(mycounts) > 0),] # Remove 0S

> mylength<-read.table('gene_length_NOISeq.txt', header=FALSE)

> myfactors = data.frame(condition = c("CB","CB","CB","PB","PB","PB","PB"), replicate =
c("CB1","CB2","CB3","PB1","PB2","PB3","PB4"))

> mydata2 = readData(mycounts, factors = myfactors, length=mylength)

> mydata2corr2 = ARSyNseq(mydata2, factor = "condition", batch = FALSE, norm = "tmm",  logtransf =
FALSE) ## remove batch effect

> mynoiseq = noiseqbio(mydata2corr2, k = 0.5, norm = "rpkm", factor = "condition", lc = 1,
random.seed=12345, filter=1, cv.cutoff = 50)


###############################################################################
################ GO enrichment analysis with GOSeq in R ##############################
###############################################################################

> library(goseq)

> de.genes <- scan("de_genes.txt", what=character() ) # List of differentially expressed genes

> assayed.genes <- scan("all_genes.txt", what=character() ) # List of all genes assayed in the experiment

> gene.length=scan("gene_lengths.txt", what=numeric() ) # For all genes not only the DE genes

> go.ids= read.table("goIDs.txt",header=FALSE) # Table that assign GO numbers to genes
```

```
> gene.vector = as.integer(assayed.genes %in% de.genes)  # Next the gene.vector (0 = not DE 1 = DE) was created from the gene files

> names(gene.vector) = assayed.genes # Add names to gene.vector

> pwf=nullp(gene.vector,bias.data=gene.length) # Calculate  Probability Weighting Function (PWF)

> GO.wall=goseq(pwf,gene2cat=go.ids, use_genes_without_cat=TRUE) # Calculate over and under expressed GO categories among DE genes with the wallenius approximation

> FDR_over <-p.adjust(GO.wall$over_represented_pvalue,method="BH") # Calculate FDR values for over_represented FDR
> FDR_under <-p.adjust(GO.wall$under_represented_pvalue,method="BH") # Calculate FDR values for under_represented FDR

> merged.data <- cbind(GO.wall,FDR_over,FDR_under) # Adds FDR values to the table

> write.table(merged.data,"GO_enrichment_Results.txt") # Last column represent the FDR values

> enriched.GO=GO.wall$category[p.adjust(GO.wall$over_represented_pvalue,method="BH")<.05] # Gives list of over represented GO numbers

> write.table(enriched.GO,"GO_over_represented05FDR.txt", row.names=F, col.names=F)

> under.GO=GO.wall$category[p.adjust(GO.wall$under_represented_pvalue,method="BH")<.05]

> write.table(under.GO,"GO_under_represented05FDR.txt", row.names=F, col.names=F)


######################################################################################
############### Kraken analysis #######################################################
######################################################################################

$ ./kraken --db Oral_microbiome/ --threads 35 --fastq-input --gzip-compressed --only-classified-output --output Sample_1.txt --paired Sample_1_R1.fastq.gz Sample_1_R2.fastq.gz

$ ./kraken-mpa-report --db Oral_microbiome/ Sample_1.txt > Sample_1.tab

$ python merge_metaphlan_tables.py Sample_1.tab  .... Sample_NSorted.tab > merged_tables_kraken.txt

$ cut -f2,3 Sample_1.txt > Sample_1.in

$ ./ImportTaxonomy.pl Sample_1.in -o Sample_1.html ###### Generates figure
```

```
#######################################################################################
################### Multiplex boxplot in R ##################################################
#######################################################################################


library(ggplot2)
df.m <- read.table("boxplot_all.txt", header=T)
p <- ggplot(data = df.m, aes(x=Label, y=value))
p <- p + geom_boxplot(aes(fill = variable))
p <- p + ylim(0, 100) # scales y-axis from 0 to 100
# if you want color for points replace group with colour=Label
p <- p + geom_point(aes(y=value, group=variable), position = position_dodge(width=0.75))
p <- p + facet_wrap( ~ Label, scales="free")
p <- p + xlab("x-axis") + ylab("y-axis") + ggtitle("Title")
p <- p + guides(fill=guide_legend(title="Legend_Title"))
p

#######################################################################################
############### Non-parametric multiple comparison Kruskal-Wallis analysis in R ###################
#######################################################################################

library(agricolae)

mydata<-read.table('mydata.txt', header= TRUE)

attach(mydata)

comparison<-kruskal(value,concentration, alpha=0.05, group=FALSE, p.adj=c("fdr"))

comparison



#######################################################################################
################## Two-way ANOVA interaction test  in R #########################################
#######################################################################################

        dat<-read.table('boxplot_all_ANOVA.txt', header=TRUE)
        plot(value ~ Label + variable, data=dat)

Hit <Return> to see next plot:

## Save Plots

        interaction.plot(dat$Label, dat$variable, dat$value)
        interaction.plot(dat$variable, dat$Label, dat$value)
        results = lm(value ~ Label + variable + Label*variable, data=dat)
        anova(results)

#######################################################################################
```
