# Supplementary material for: Potassium is a key signal in host-microbiome dysbiosis in periodontitis
Source: PLoS Pathog. 2017 Jun 20;13(6):e1006457. doi: 10.1371/journal.ppat.1006457 (PMC5493431; doi:10.1371/journal.ppat.1006457)
Supplement: S1 Fig — NOISeq qualitity analysis of the sequencing libraries. A) Saturation plot for protein-coding genes for all samples. B) Sensitivity plot. Percentage of features having more than 0, 1, 2, 5 and 10 counts per million (CPM). (PDF) [file ppat.1006457.s002.pdf]

Sequencing depth & Expression quantification

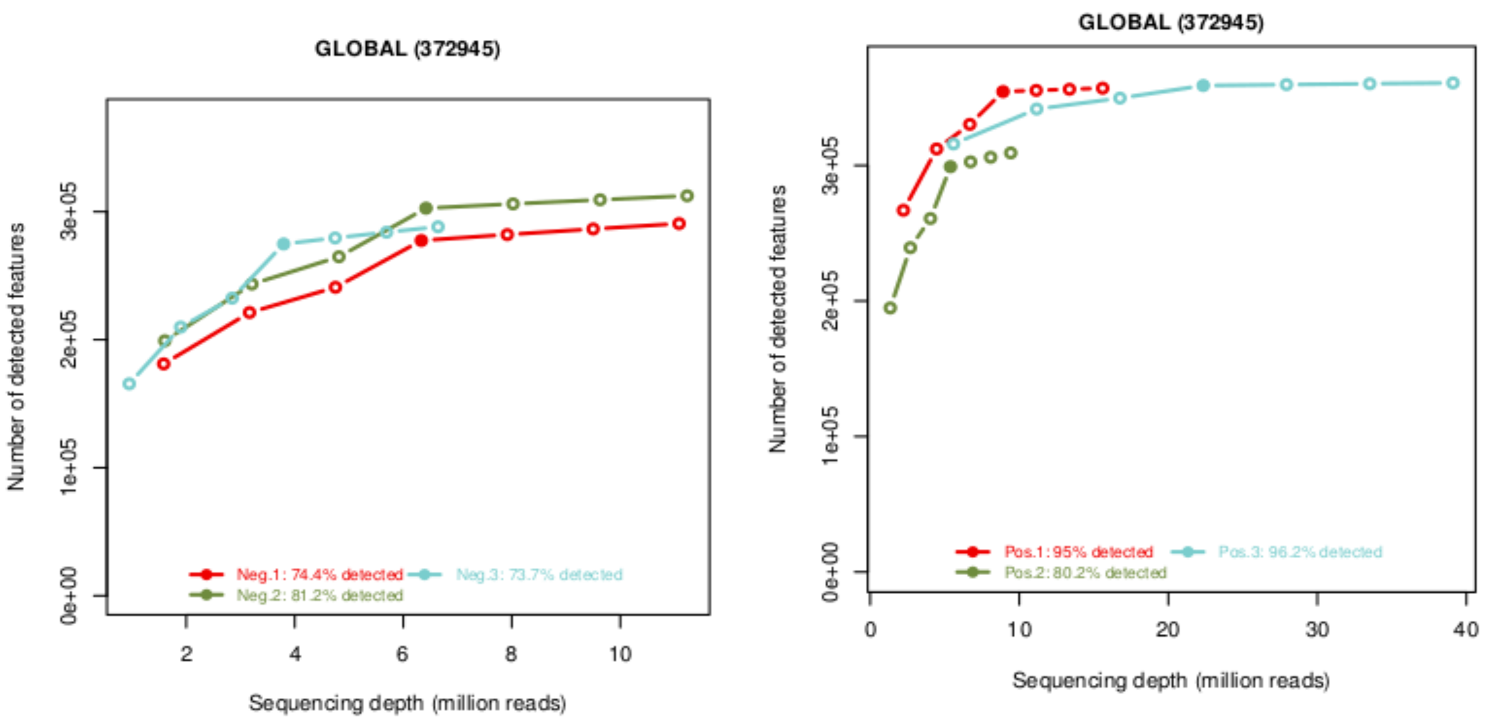

A) Saturation plot for protein-coding genes for all samples

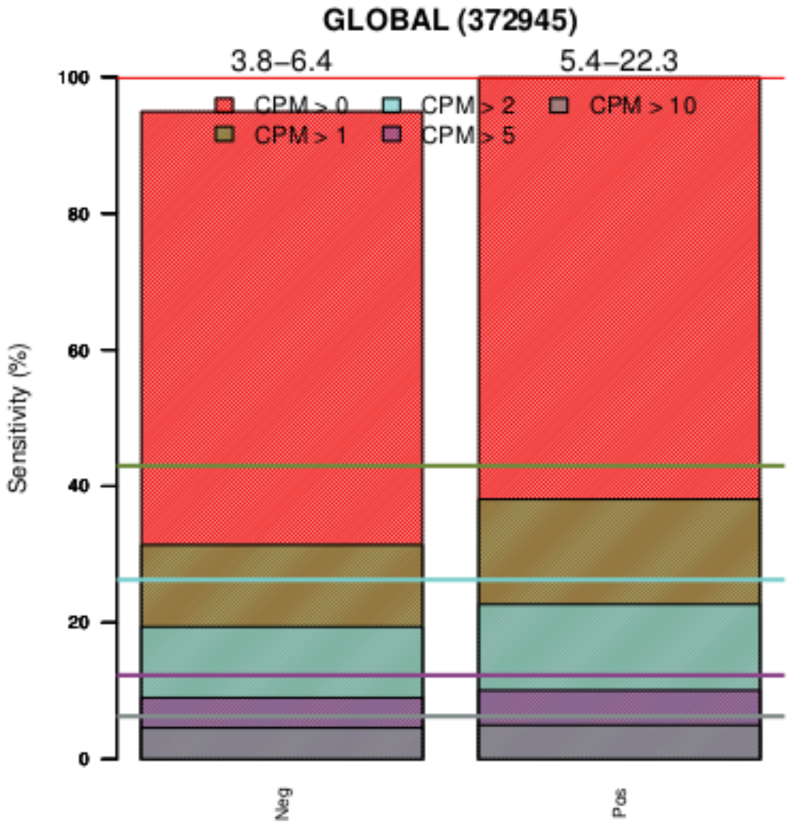

B) Sensitivity plot. Percentage of features having more than 0,1,2,5 and 10 counts per million (CPM)
