# Supplementary material for: Potassium is a key signal in host-microbiome dysbiosis in periodontitis
Source: PLoS Pathog. 2017 Jun 20;13(6):e1006457. doi: 10.1371/journal.ppat.1006457 (PMC5493431; doi:10.1371/journal.ppat.1006457)
Supplement: S2 Fig — A) 0mM No plaque 20x B) 50mM K+ No Plaque 20x C) 0mM K+ Plaque 20x D) 50mM K+ Plaque 20x. Key: SC, Stratum corneum, BC, Basal cells, MM, Microporous membrane. (PDF) [file ppat.1006457.s003.pdf]

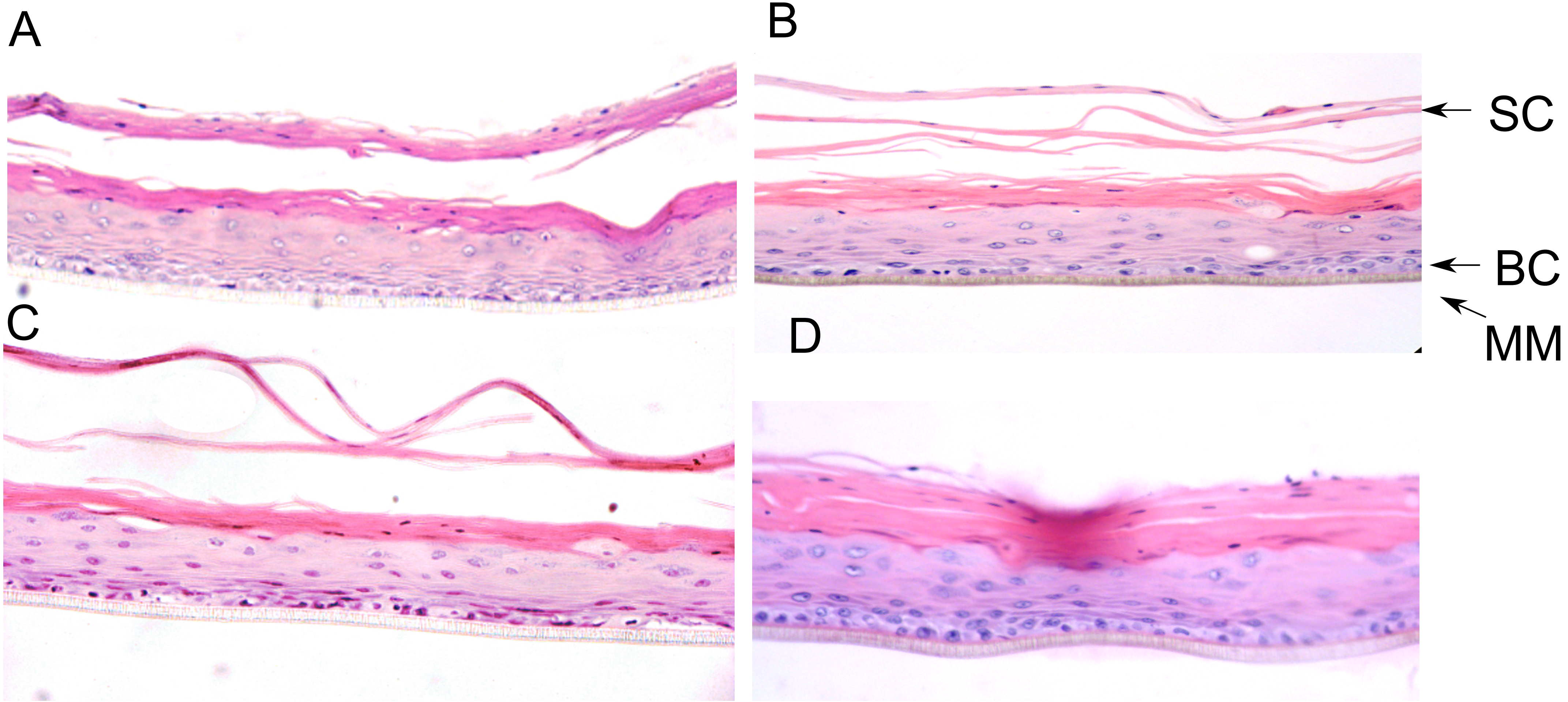

S2 Fig. Haematoxylin-eosin stained sections of the 3D gingival tissue model.
A) 0mM No plaque 20x,
B) 50mM No Plaque 20x
C) 0mM Plaque 20x
D) 50mM Plaque 20x

Key: SC, Stratum corneum, BC, Basal cells, MM, Microporous membrane
