# Supplementary material for: Potassium is a key signal in host-microbiome dysbiosis in periodontitis
Source: PLoS Pathog. 2017 Jun 20;13(6):e1006457. doi: 10.1371/journal.ppat.1006457 (PMC5493431; doi:10.1371/journal.ppat.1006457)
Supplement: S3 Fig — Brightly fluorescent cell-associated bacteria seen with the EUB338 probe. Green autofluorescence background from the tissue. A) 0 K+ added. B) 50mM K+ added. Magnification, ×400. (PDF) [file ppat.1006457.s004.pdf]

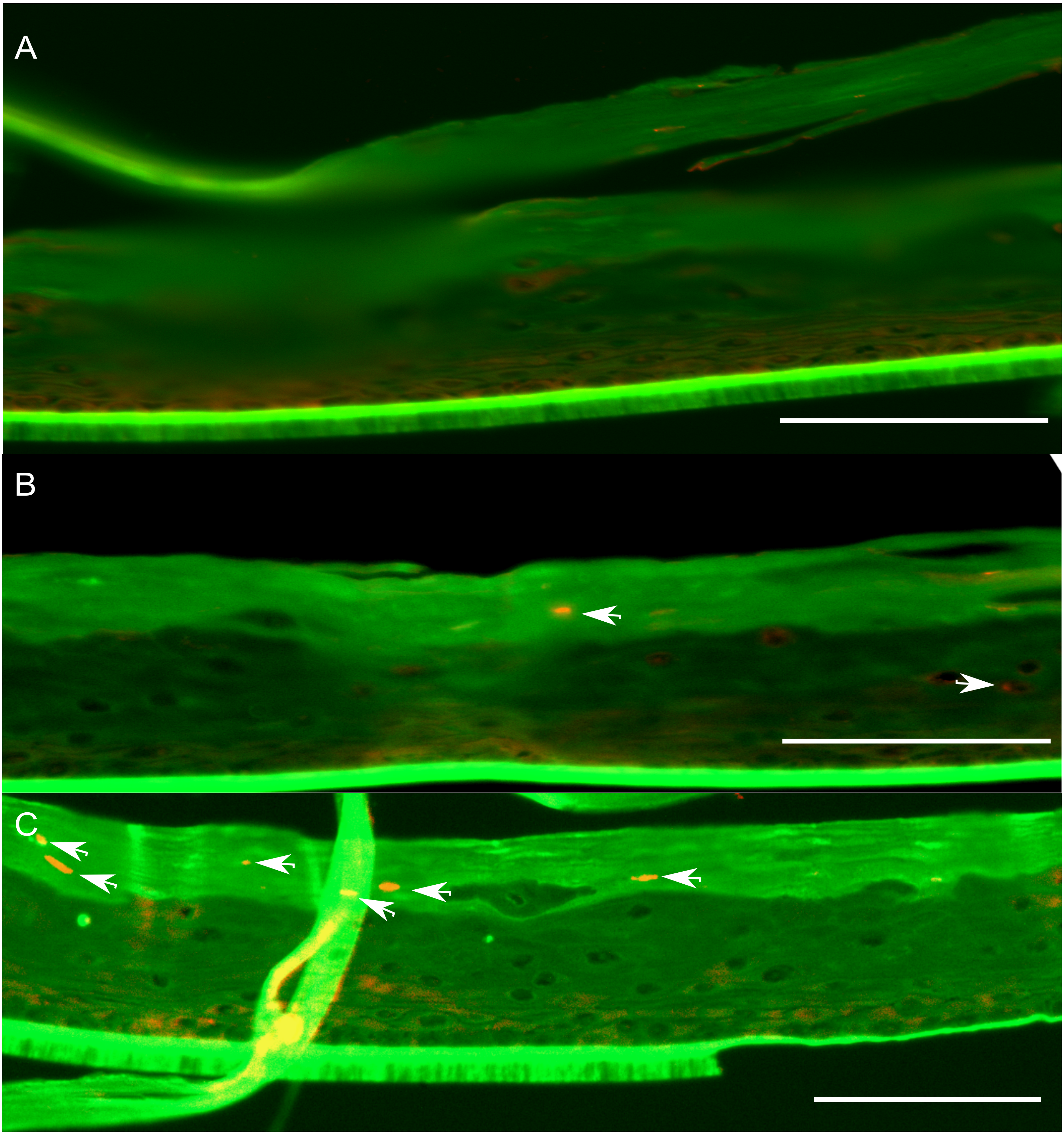

S3 Fig. FISH of tissue culture invasion. Brightly fluorescent cell-associated bacteria seen with the EUB338 probe.

Green autofluorescence background from the tissue.

A) 0 K+ added + No plaque.

B) 0 K+ added + plaque.

C) 50mM K+ added + plaque.

Scale bar = 50 µm. Magnification 400x
