# Supplementary material for: Potassium is a key signal in host-microbiome dysbiosis in periodontitis
Source: PLoS Pathog. 2017 Jun 20;13(6):e1006457. doi: 10.1371/journal.ppat.1006457 (PMC5493431; doi:10.1371/journal.ppat.1006457)
Supplement: S4 Fig — A three-dimensional multilayered gingival tissue model with cornified apical layers (EpiGingival, MatTek Corporation) was used to assess the effect of different concentrations of K+ and bacteria on the profiles of expression of different cytokines. Cytokine expression was measured by Luminex under different K+ concentrations. Box plots show the values of observed concentrations in the media of the different cytokines assayed. (PDF) [file ppat.1006457.s005.pdf]

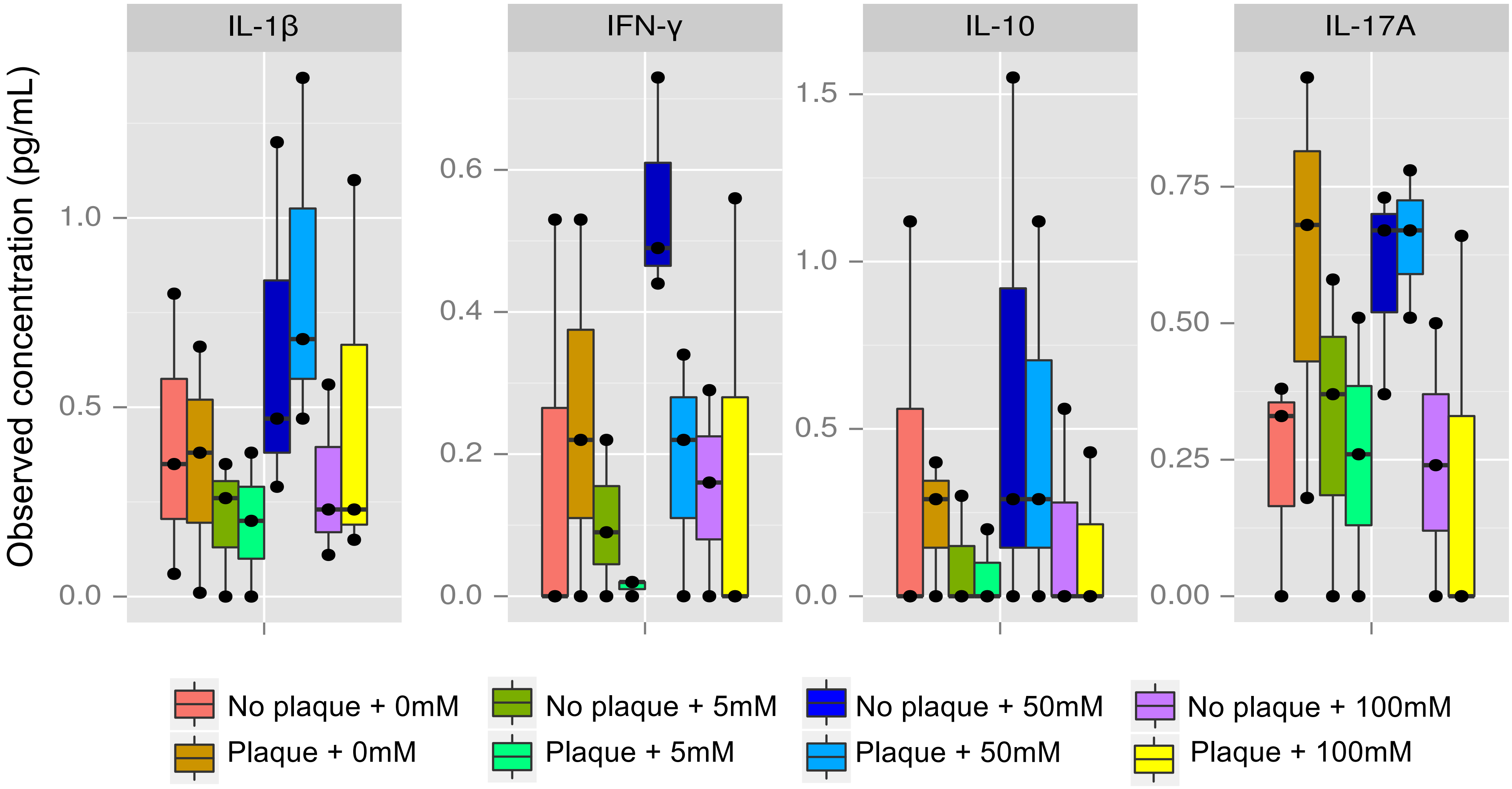

S4 Fig. **Effect of potassium concentration on gingival cytokine expression.**
A three-dimensional multilayered gingival tissue model with cornified apical layers (EpiGingival, MatTek Corporation) was used to assess the effect of different concentrations of K+ and bacteria on the profiles of expression of different cytokines. Cytokine expression was measured by Luminex under different K+ concentrations.

Box plots show the values of observed concentrations in the media of the different cytokines assayed.
