# Supplementary material for: Potassium is a key signal in host-microbiome dysbiosis in periodontitis
Source: PLoS Pathog. 2017 Jun 20;13(6):e1006457. doi: 10.1371/journal.ppat.1006457 (PMC5493431; doi:10.1371/journal.ppat.1006457)
Supplement: S5 Fig — A) Interaction plots displaying the levels of one factor on the x-axis and the mean response on the y-axis for all cytokines analyzed. B) Interaction plots displaying the levels of one factor on the x-axis and the mean response on the y-axis for IL-6. C) Interaction plots displaying the levels of one factor on the x-axis and the mean response on the y-axis for TNF-α. (PDF) [file ppat.1006457.s006.pdf]

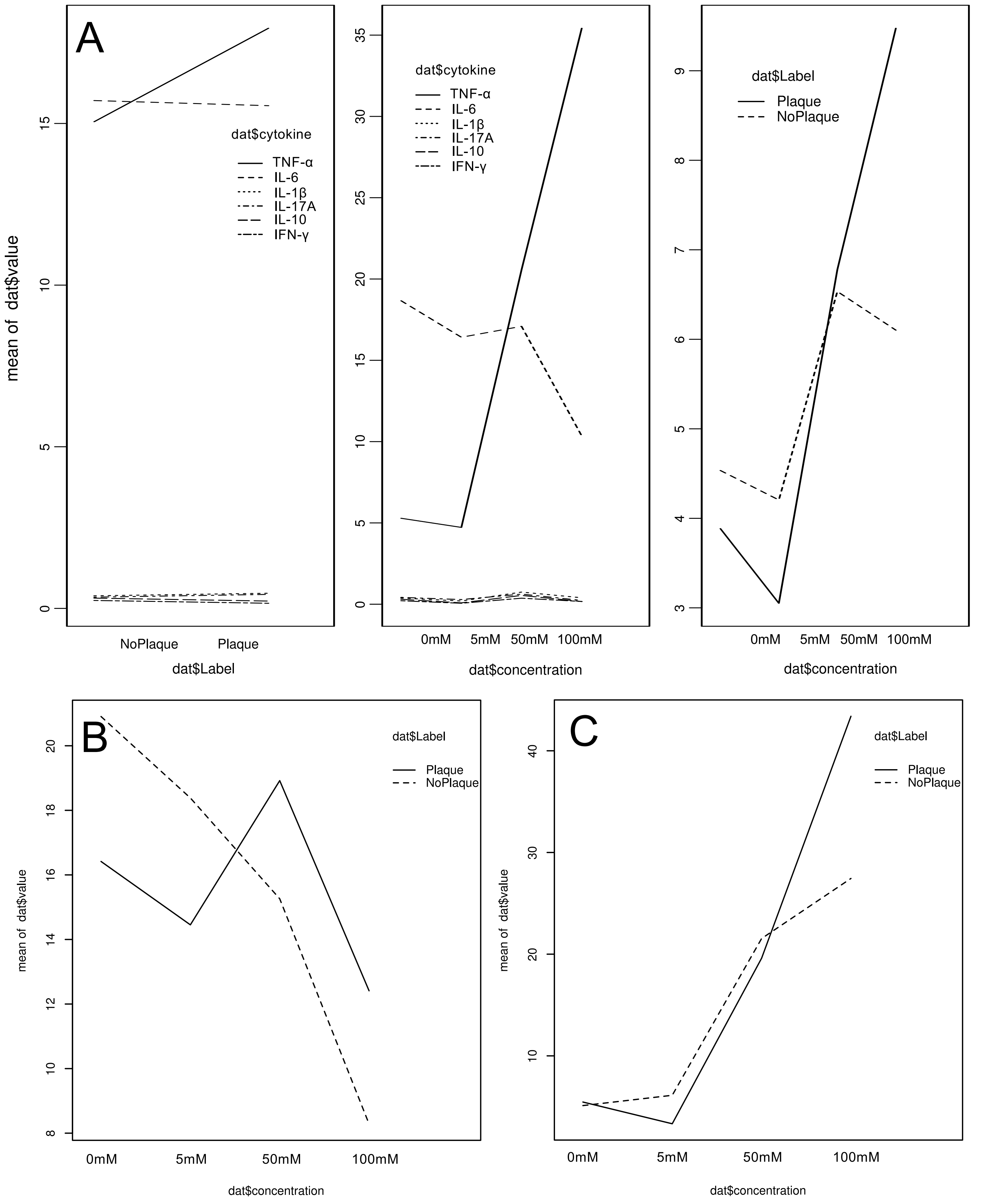

S5 Fig. **Interaction analysis Labels (plaque/no plaque) and potassium concentration.**
A) Interaction plots displaying the levels of one factor on the x-axis and the mean response on the y-axis for all cytokines analyzed.
B) Interaction plots displaying the levels of one factor on the x-axis and the mean response on the y-axis for IL-6.
C) Interaction plots displaying the levels of one factor on the x-axis and the mean response on the y-axis for TNF-α.
