# Supplementary material for: Potassium is a key signal in host-microbiome dysbiosis in periodontitis
Source: PLoS Pathog. 2017 Jun 20;13(6):e1006457. doi: 10.1371/journal.ppat.1006457 (PMC5493431; doi:10.1371/journal.ppat.1006457)
Supplement: S6 Fig — A) Boxplots showing values based on label (plaque/no plaque) and potassium concentrations. B) Interaction plots displaying the levels of one factor on the x-axis and the mean response on the y-axis. (PDF) [file ppat.1006457.s007.pdf]

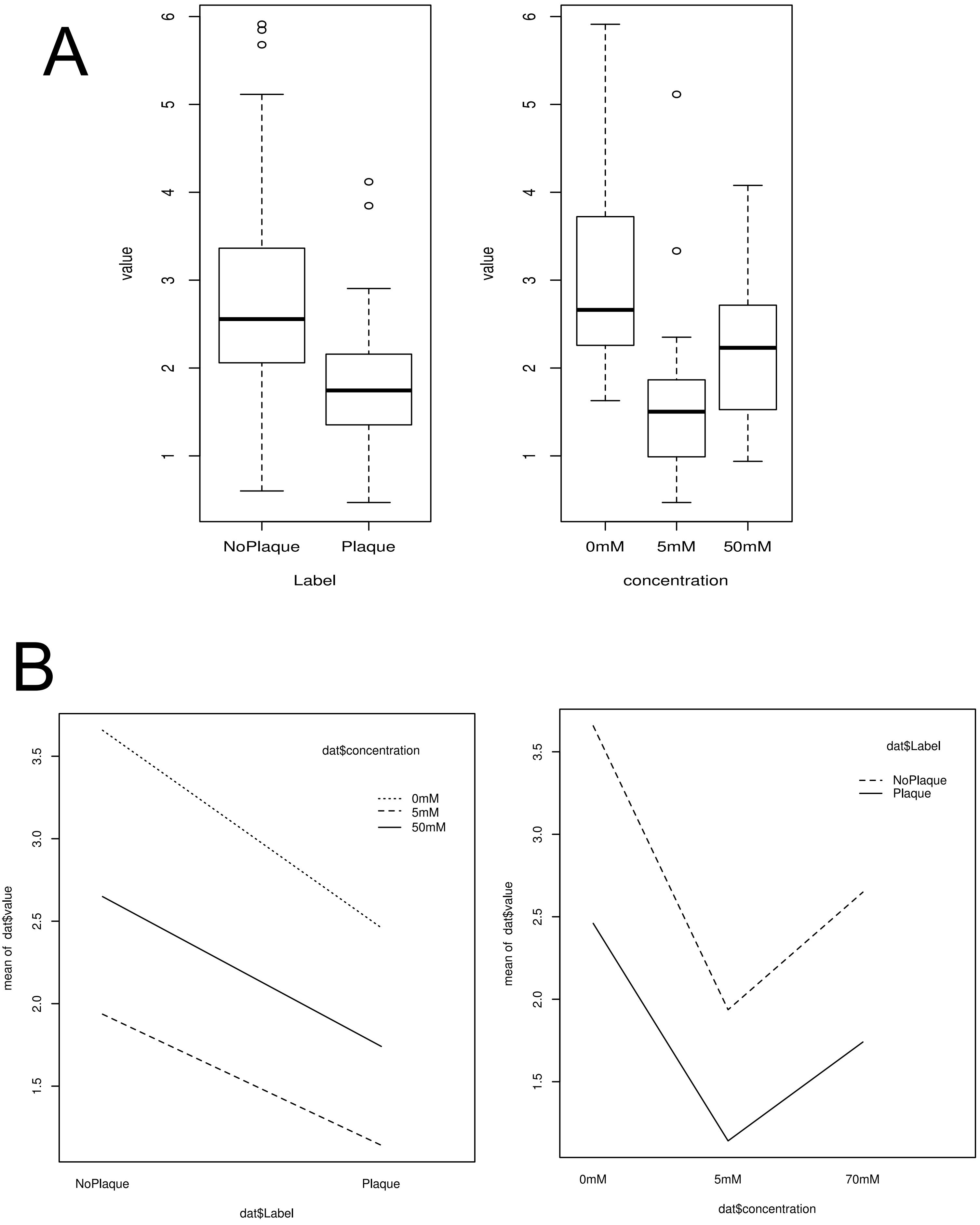

S6 Fig. **Interaction analysis Labels (plaque/no plaque) and potassium concentration.**
A) Boxplots showing values based on label (plaque/no plaque) and potassium concentrations.
B) Interaction plots displaying the levels of one factor on the x-axis and the mean response on the y-axis.
