# Supplementary figures and images for: Potassium is a key signal in host-microbiome dysbiosis in periodontitis
Source: PLoS Pathog. 2017 Jun 20;13(6):e1006457. doi: 10.1371/journal.ppat.1006457 (PMC5493431; doi:10.1371/journal.ppat.1006457)

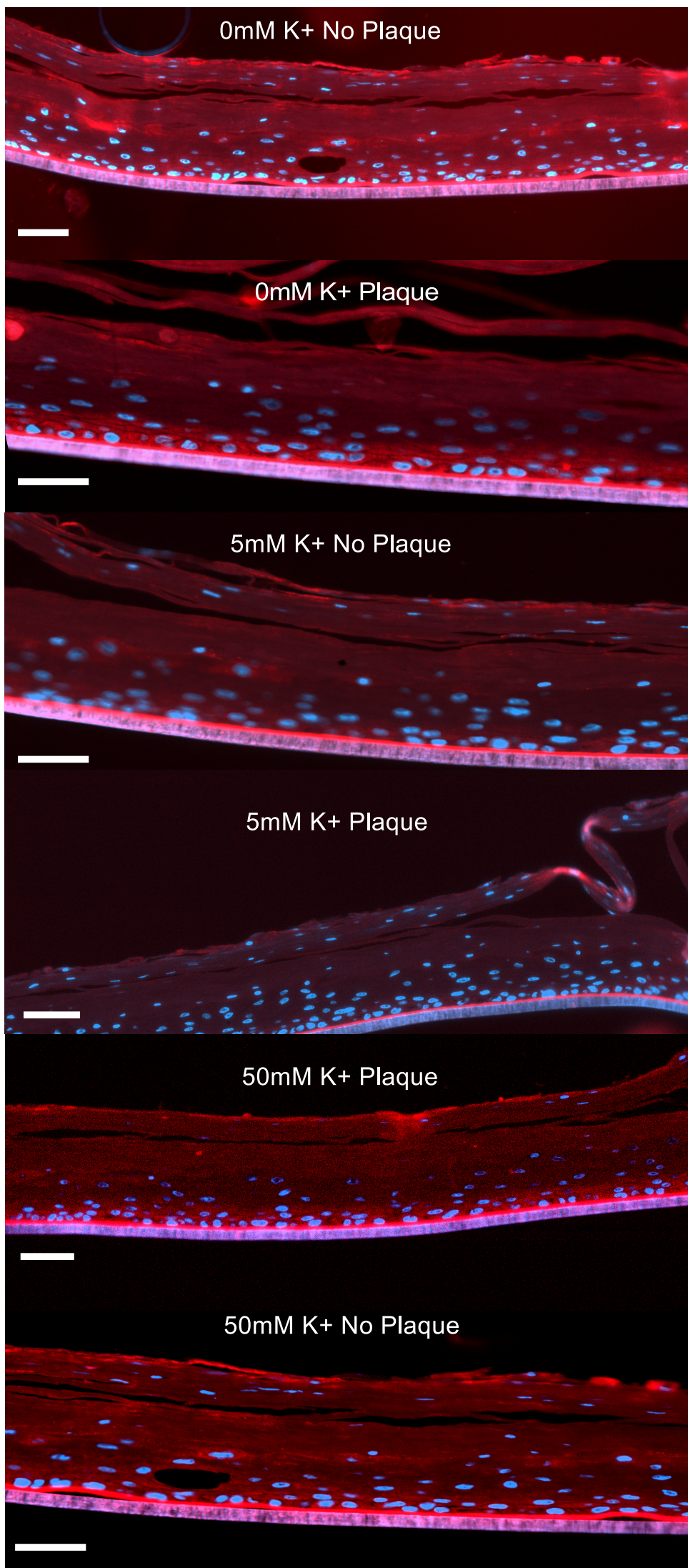

Supplement: S7 Fig — Representative images of all treatments performed. We used the red fluorescence for hBD-3 expression (Alexa Fluor 594) and blue fluorescence (DAPI) to stain cells' nuclei. Scale bars = 25 μm. (PDF) [file ppat.1006457.s008.pdf]
