# Supplementary material for: Potassium is a key signal in host-microbiome dysbiosis in periodontitis
Source: PLoS Pathog. 2017 Jun 20;13(6):e1006457. doi: 10.1371/journal.ppat.1006457 (PMC5493431; doi:10.1371/journal.ppat.1006457)
Supplement: S1 Table — 50mM of K+ were added to dental plaque on saliva and incubated for 3 hours at 37°C in anaerobic conditions. GO terms enrichment analysis was performed as described in the Methods section. (PDF) [file ppat.1006457.s009.pdf]

S1 Table. Summary of Molecular Function Gene Ontolology (GO) terms enriched in the presence of potassium. 50mM of K+ were added to dental plaque on saliva and incubateed for 3 hours at 37°C in anaerobic conditions. GO terms enrichment analysis was performed as described in the Methods section.

**GO terms Over-represented in the presence of 50mM K+ added**	
**Gene Ontology (GO) ID**	**Description**
GO:0003711	transcription elongation regulator activity
GO:0043365	[formate-C-acetyltransferase]-activating enzyme activity
GO:0030604	1-deoxy-D-xylulose-5-phosphate reductoisomerase activity
GO:0008661	1-deoxy-D-xylulose-5-phosphate synthase activity
GO:0016308	1-phosphatidylinositol-4-phosphate 5-kinase activity
GO:0050518	2-C-methyl-D-erythritol 4-phosphate cytidylyltransferase activity
GO:0047553	2-oxoglutarate synthase activity
GO:0008676	3-deoxy-8-phosphooctulonate synthase activity
GO:0008690	3-deoxy-manno-octulosonate cytidylyltransferase activity
GO:0019143	3-deoxy-manno-octulosonate-8-phosphatase activity
GO:0003864	3-methyl-2-oxobutanoate hydroxymethyltransferase activity
GO:0004315	3-oxoacyl-[acyl-carrier-protein] synthase activity
GO:0004134	4-alpha-glucanotransferase activity
GO:0008703	5-amino-6-(5-phosphoribosylamino)uracil reductase activity
GO:0003872	6-phosphofructokinase activity
GO:0008780	acyl-[acyl-carrier-protein]-UDP-N-acetylglucosamine O-acyltransferase activity
GO:0000774	adenyl-nucleotide exchange factor activity
GO:0004017	adenylate kinase activity
GO:0004019	adenylosuccinate synthase activity
GO:0004813	alanine-tRNA ligase activity
GO:0004560	alpha-L-fucosidase activity
GO:0004177	aminopeptidase activity
GO:0004814	arginine-tRNA ligase activity
GO:0004067	asparaginase activity
GO:0004816	asparagine-tRNA ligase activity
GO:0005524	ATP binding
GO:0018522	benzoyl-CoA reductase activity
GO:0004563	beta-N-acetylhexosaminidase activity
GO:0005488	binding
GO:0004076	biotin synthase activity
GO:0004084	branched-chain-amino-acid transaminase activity

GO:0003824	catalytic activity
GO:0051087	chaperone binding
GO:0008817	cob(I)yrinic acid a,c-diamide adenosyltransferase activity
GO:0048037	cofactor binding
GO:0004124	cysteine synthase activity
GO:0004127	cytidylate kinase activity
GO:0008834	di-trans,poly-cis-decaprenylcistransferase activity
GO:0008835	diaminohydroxyphosphoribosylaminopyrimidine deaminase activity
GO:0047850	diaminopimelate dehydrogenase activity
GO:0004149	dihydrolipoyllysine-residue succinyltransferase activity
GO:0004156	dihydropteroate synthase activity
GO:0047334	diphosphate-fructose-6-phosphate 1-phosphotransferase activity
GO:0003917	DNA topoisomerase type I activity
GO:0003918	DNA topoisomerase type II (ATP-hydrolyzing) activity
GO:0003899	DNA-directed RNA polymerase activity
GO:0008831	dTDP-4-dehydrorhamnose reductase activity
GO:0004170	dUTP diphosphatase activity
GO:0004311	farnesyltranstransferase activity
GO:0010181	FMN binding
GO:0008861	formate C-acetyltransferase activity
GO:0004329	formate-tetrahydrofolate ligase activity
GO:0042132	fructose 1,6-bisphosphate 1-phosphatase activity
GO:0004332	fructose-bisphosphate aldolase activity
GO:0004333	fumarate hydratase activity
GO:0050577	GDP-L-fucose synthase activity
GO:0004342	glucosamine-6-phosphate deaminase activity
GO:0008877	glucose-1-phosphatase activity
GO:0008879	glucose-1-phosphate thymidylyltransferase activity
GO:0004354	glutamate dehydrogenase (NADP+) activity
GO:0030409	glutamate formimidoyltransferase activity
GO:0004818	glutamate-tRNA ligase activity
GO:0004819	glutamine-tRNA ligase activity
GO:0004365	glyceraldehyde-3-phosphate dehydrogenase (NAD+) (phosphorylating) activity
GO:0008890	glycine C-acetyltransferase activity
GO:0005525	GTP binding
GO:0004416	hydroxyacylglutathione hydrolase activity
GO:0004418	hydroxymethylbilane synthase activity

GO:0005315	inorganic phosphate transmembrane transporter activity
GO:0004512	inositol-3-phosphate synthase activity
GO:0005216	ion channel activity
GO:0005381	iron ion transmembrane transporter activity
GO:0030060	L-malate dehydrogenase activity
GO:0008743	L-threonine 3-dehydrogenase activity
GO:0015129	lactate transmembrane transporter activity
GO:0008928	mannose-1-phosphate guanylyltransferase (GDP) activity
GO:0008235	metalloexopeptidase activity
GO:0004478	methionine adenosyltransferase activity
GO:0050097	methylaspartate mutase activity
GO:0004492	methylmalonyl-CoA decarboxylase activity
GO:0004494	methylmalonyl-CoA mutase activity
GO:0008168	methyltransferase activity
GO:0008747	N-acetylneuraminate lyase activity
GO:0008137	NADH dehydrogenase (ubiquinone) activity
GO:0000309	nicotinamide-nucleotide adenylyltransferase activity
GO:0042279	nitrite reductase (cytochrome, ammonia-forming) activity
GO:0050421	nitrite reductase (NO-forming) activity
GO:0003676	nucleic acid binding
GO:0008171	O-methyltransferase activity
GO:0004648	O-phospho-L-serine:2-oxoglutarate aminotransferase activity
GO:0008948	oxaloacetate decarboxylase activity
GO:0004591	oxoglutarate dehydrogenase (succinyl-transferring) activity
GO:0042586	peptide deformylase activity
GO:0003755	peptidyl-prolyl cis-trans isomerase activity
GO:0004605	phosphatidate cytidylyltransferase activity
GO:0004609	phosphatidylserine decarboxylase activity
GO:0008963	phospho-N-acetylmuramoyl-pentapeptide-transferase activity
GO:0004612	phosphoenolpyruvate carboxykinase (ATP) activity
GO:0004618	phosphoglycerate kinase activity
GO:0004634	phosphopyruvate hydratase activity
GO:0004639	phosphoribosylaminoimidazolesuccinocarboxamide synthase activity
GO:0004641	phosphoribosylformylglycinamidine cyclo-ligase activity
GO:0004644	phosphoribosylglycinamide formyltransferase activity
GO:0004654	polyribonucleotide nucleotidyltransferase activity
GO:0004655	porphobilinogen synthase activity

GO:0046026	precorrin-4 C11-methyltransferase activity
GO:0016994	precorrin-6A reductase activity
GO:0005515	protein binding
GO:0008565	protein transporter activity
GO:0046933	proton-transporting ATP synthase activity, rotational mechanism
GO:0046961	proton-transporting ATPase activity, rotational mechanism
GO:0004743	pyruvate kinase activity
GO:0004746	riboflavin synthase activity
GO:0004748	ribonucleoside-diphosphate reductase activity, thioredoxin disulfide as acceptor
GO:0050262	ribosylnicotinamide kinase activity
GO:0003723	RNA binding
GO:0016987	sigma factor activity
GO:0003697	single-stranded DNA binding
GO:0016852	sirohydrochlorin cobaltochelatase activity
GO:0003735	structural constituent of ribosome
GO:0004775	succinate-CoA ligase (ADP-forming) activity
GO:0009029	tetraacyldisaccharide 4'-kinase activity
GO:0016740	transferase activity
GO:0016746	transferase activity, transferring acyl groups
GO:0003746	translation elongation factor activity
GO:0003743	translation initiation factor activity
GO:0003747	translation release factor activity
GO:0004807	triose-phosphate isomerase activity
GO:0004808	tRNA (5-methylaminomethyl-2-thiouridylate)-methyltransferase activity
GO:0009019	tRNA (guanine-N1-)-methyltransferase activity
GO:0052381	tRNA dimethylallyltransferase activity
GO:0009034	tryptophanase activity
GO:0008759	UDP-3-O-[3-hydroxymyristoyl] N-acetylglucosamine deacetylase activity
GO:0003978	UDP-glucose 4-epimerase activity
GO:0008765	UDP-N-acetylmuramoylalanyl-D-glutamate-2,6-diaminopimelate ligase activity
GO:0050511	undecaprenyldiphospho-muramoylpentapeptide beta-N acetylglucosaminyltransferase activity
GO:0004849	uridine kinase activity

GO terms Under-represented in the presence of 50mM K+ added	
GO:0003988	acetyl-CoA C-acyltransferase activity
GO:0003985	acetyl-CoA C-acetyltransferase activity
GO:0004871	signal transducer activity
GO:0030151	molybdenum ion binding
GO:0008706	6-phospho-beta-glucosidase activity
GO:0008422	beta-glucosidase activity
GO:0004467	long-chain fatty acid-CoA ligase activity
GO:0008692	3-hydroxybutyryl-CoA epimerase activity
GO:0004300	enoyl-CoA hydratase activity
GO:0003857	3-hydroxyacyl-CoA dehydrogenase activity
GO:0004334	fumarylacetoacetase activity
GO:0008260	3-oxoacid CoA-transferase activity
GO:0008748	N-ethylmaleimide reductase activity
GO:0004364	glutathione transferase activity
GO:0009039	urease activity
GO:0003992	N2-acetyl-L-ornithine:2-oxoglutarate 5-aminotransferase activity
GO:0008955	peptidoglycan glycosyltransferase activity
GO:0008984	protein-glutamate methylesterase activity
GO:0008234	cysteine-type peptidase activity
GO:0047121	isoquinoline 1-oxidoreductase activity
GO:0016041	glutamate synthase (ferredoxin) activity
GO:0008982	protein-N(PI)-phosphohistidine-sugar phosphotransferase activity
GO:0003960	NADPH:quinone reductase activity
GO:0008726	alkanesulfonate monooxygenase activity
GO:0004029	aldehyde dehydrogenase (NAD) activity
GO:0008827	cytochrome o ubiquinol oxidase activity
GO:0004096	catalase activity
GO:0047905	fructose-6-phosphate phosphoketolase activity
GO:0004485	methylcrotonoyl-CoA carboxylase activity
GO:0018849	muconate cycloisomerase activity
GO:0004016	adenylate cyclase activity
GO:0004165	dodecenoyl-CoA delta-isomerase activity
GO:0008470	isovaleryl-CoA dehydrogenase activity
GO:0050567	glutaminyl-tRNA synthase (glutamine-hydrolyzing) activity
GO:0004066	asparagine synthase (glutamine-hydrolyzing) activity
GO:0019154	glycolate dehydrogenase activity

GO:0004108	citrate (Si)-synthase activity
GO:0008866	fructuronate reductase activity
GO:0008876	quinoprotein glucose dehydrogenase activity
GO:0050193	phosphoketolase activity
GO:0004131	cytosine deaminase activity
GO:0004042	acetyl-CoA:L-glutamate N-acetyltransferase activity
GO:0008080	N-acetyltransferase activity
GO:0008477	purine nucleosidase activity
GO:0008811	chloramphenicol O-acetyltransferase activity
GO:0008679	2-hydroxy-3-oxopropionate reductase activity
GO:0004777	succinate-semialdehyde dehydrogenase (NAD+) activity
GO:0008812	choline dehydrogenase activity
GO:0047265	poly(glycerol-phosphate) alpha-glucosyltransferase activity
GO:0004555	alpha,alpha-trehalase activity
GO:0008933	lytic transglycosylase activity
GO:0003879	ATP phosphoribosyltransferase activity
GO:0016223	beta-alanine-pyruvate transaminase activity
GO:0000908	taurine dioxygenase activity
GO:0004450	isocitrate dehydrogenase (NADP+) activity
GO:0004022	alcohol dehydrogenase (NAD) activity
GO:0008442	3-hydroxyisobutyrate dehydrogenase activity
GO:0004357	glutamate-cysteine ligase activity
GO:0008700	4-hydroxy-2-oxoglutarate aldolase activity
GO:0008677	2-dehydropantoate 2-reductase activity
GO:0008556	potassium-transporting ATPase activity
GO:0004471	malate dehydrogenase (decarboxylating) (NAD+) activity
GO:0050566	asparaginyl-tRNA synthase (glutamine-hydrolyzing) activity
GO:0018623	benzoate 1,2-dioxygenase activity
GO:0004358	glutamate N-acetyltransferase activity
GO:0009018	sucrose phosphorylase activity
GO:0018578	protocatechuate 3,4-dioxygenase activity
GO:0003868	4-hydroxyphenylpyruvate dioxygenase activity
GO:0008802	betaine-aldehyde dehydrogenase activity
GO:0003849	3-deoxy-7-phosphoheptulonate synthase activity
GO:0004015	adenosylmethionine-8-amino-7-oxononanoate transaminase activity
GO:0031071	cysteine desulfurase activity
GO:0008324	cation transmembrane transporter activity

GO:0008514	organic anion transmembrane transporter activity
GO:0017057	6-phosphogluconolactonase activity
GO:0004622	lysophospholipase activity
GO:0003942	N-acetyl-gamma-glutamyl-phosphate reductase activity
GO:0009025	tagatose-bisphosphate aldolase activity
GO:0030729	acetoacetate-CoA ligase activity
GO:0015079	potassium ion transmembrane transporter activity
