# Supplementary material for: Potassium is a key signal in host-microbiome dysbiosis in periodontitis
Source: PLoS Pathog. 2017 Jun 20;13(6):e1006457. doi: 10.1371/journal.ppat.1006457 (PMC5493431; doi:10.1371/journal.ppat.1006457)
Supplement: S3 Table — Kruskal-Wallis analysis corrected for multiple comparisons of percentage of hemolytic actitivy and growth measured as OD600 and CFUs after 12 hours of incubation. Tables show corrected p-values. In yellow are comparisons that were statistically significant with a p-value < 0.05. (PDF) [file ppat.1006457.s011.pdf]

S3 Table. A) Total number of bacteria measured by qPCR in inocula and agar plates used on hemolytic activity of dental plaque experiments. B) p-values of Kruskal-Wallis analysis corrected for multiple comparisons. In yellow are differences that were statistically significant.

A

	Inocula	0mM	0.5mM	5mM	50mM
Patient 1	**$2.26x10^7$** $\pm3.28x10^5$	**$7.14x10^{10}$** $\pm1.18x10^9$	**$7.23x10^{10}$** $\pm8.68x10^8$	**$7.42x10^{10}$** $\pm3.28x10^8$	**$7.59x10^{10}$** $\pm1.188x10^9$
Patient 2	**$3.07x10^7$** $\pm4.03x10^6$	**$7.48x10^{10}$** $\pm8.68x10^8$	**$9.67x10^{10}$** $\pm6.56x10^8$	**$9.90x10^{10}$** $\pm3.13x10^9$	**$7.61x10^{10}$** $\pm4.29x10^9$
Patient 3	**$2.95x10^7$** $\pm1.18x10^6$	**$4.65x10^{10}$** $\pm8.68x10^8$	**$4.24x10^{10}$** $\pm8.68x10^8$	**$4.98x10^{10}$** $\pm4.02x10^8$	**$2.67x10^{10}$** $\pm1.50x10^9$

B

	Inocula	0mM	0.5mM	5mM	50mM
**Inocula**	1	0.0035	0.0000	0.0002	0.0000
**0mM**		1	0.0866	0.2984	0.0592
**0.5mM**			1	0.4234	0.1567
**5mM**				1	0.0674
**50mM**					1
