# Supplementary material for: Potassium is a key signal in host-microbiome dysbiosis in periodontitis
Source: PLoS Pathog. 2017 Jun 20;13(6):e1006457. doi: 10.1371/journal.ppat.1006457 (PMC5493431; doi:10.1371/journal.ppat.1006457)
Supplement: S5 Table — A) Total number of bacteria measured by qPCR in inocula and agar plates used on hemolytic activity of dental plaque experiments. B) p-values of Kruskal-Wallis analysis corrected for multiple comparisons. In yellow are differences that were statistically significant. (PDF) [file ppat.1006457.s013.pdf]

S5 Table. Hemolytic activity analysis. Kruskal-Wallis analysis corrected for multiple comparisons of percentage of hemolytic actitivy and growth measured as OD_{600} and CFUs after 12 hours of incubation. Tables show corrected p-values. In yellow are comparisons that were statistically significant with a p-value < 0.05.

Prevotella nigrescens ATCC 33563 Hemolytic activity values				
	0mM	0.5mM	5mM	50mM
0mM	1	0.0159	0.05	0.0332
0.5mM		1	0.3523	0.0005
5mM			1	0.0012
50mM				1

Streptococcus mitis NCTC12261 Hemolytic activity values				
	0mM	0.5mM	5mM	50mM
0mM	1	0.0006	0.0027	0.0112
0.5mM		1	0.4336	0.0000
5mM			1	0.0000
50mM				1

S3 Table. Hemolytic activity analysis. Kruskal-Wallis analysis corrected for multiple comparisons of percentage of hemolytic actitivy and growth measured as OD_{600} and CFUs after 12 hours of incubation. Tables show corrected p-values. In yellow are comparisons that were statistically significant with a p-value < 0.050.

Prevotella nigrescens ATCC 33563 OD_{600} after 12 hours of incubation				
	0mM	0.5mM	5mM	50mM
0mM	1	0.781349	0.781349	0.781349
0.5mM		1	0.781349	1
5mM			1	0.781349
50mM				1

Prevotella nigrescens NTCC 9336 OD_{600} after 12 hours of incubation				
	0mM	0.5mM	5mM	50mM
0mM	1	0.799349	0.470992	0.827564
0.5mM		1	0.470992	0.785871
5mM			1	0.470992
50mM				1

Streptococcus mitis NCTC12261 OD_{600} after 12 hours of incubation				
	0mM	0.5mM	5mM	50mM
0mM	1	1	0.075293	0.075293
0.5mM		1	0.075293	0.075293
5mM			1	0.041412
50mM				1

S3 Table. Hemolytic activity analysis. Kruskal-Wallis analysis corrected for multiple comparisons of percentage of hemolytic actitivy and growth measured as OD_{600} and CFUs after 12 hours of incubation. Tables show corrected p-values. In yellow are comparisons that were statistically significant with a p-value < 0.050.

Prevotella nigrescens **ATCC 33563 CFUs after 12 hours of incubation**				
	0mM	0.5mM	5mM	50mM
0mM	1	0.7813	0.7813	0.7813
0.5mM		1	0.7813	1.0000
5mM			1	0.7813
50mM				1

Prevotella nigrescens **NTCC 9336 CFUs after 12 hours of incubation**				
	0mM	0.5mM	5mM	50mM
0mM	1	0.7993	0.8276	0.4710
0.5mM		1	0.4710	0.7859
5mM			1	0.4710
50mM				1

Streptococcus mitis **NCTC12261 CFUs after 12 hours of incubation**				
	0mM	0.5mM	5mM	50mM
0mM	1	1.0000	0.0753	0.0753
0.5mM		1	0.0753	0.0753
5mM			1	0.0414
50mM				1
