# Supplementary material for: Potassium is a key signal in host-microbiome dysbiosis in periodontitis
Source: PLoS Pathog. 2017 Jun 20;13(6):e1006457. doi: 10.1371/journal.ppat.1006457 (PMC5493431; doi:10.1371/journal.ppat.1006457)
Supplement: S6 Table — Kruskal-Wallis analysis corrected for multiple comparisons. In yellow are comparisons that were statistically significant with corrected p-value < 0.05. In green are comparisons that were statistically significant with corrected p-value < 0.1. NP, no plaque. P, plaque. (PDF) [file ppat.1006457.s014.pdf]

S6 Table. Differences in cytokine expression due to K+ concentration and presence or absence of dental plaque. Kruskal-Wallis analysis corrected for multiple comparisons. In yellow are comparisons that were statistically significant with corrected p-value < 0.05. In green are comparisons that were statistically significant with corrected p-value < 0.1. NP, no plaque. P, plaque.

	NP+0mM IFN-γ	NP+5mM IFN-γ	NP+50mM IFN-γ	NP+100mM IFN-γ	P+0mM IFN-γ	P+5mM IFN-γ	P+50mM IFN-γ	P+100mM IFN-γ
NP+0mM IFN-γ	1	0.9772	0.6904	0.9772	0.9772	0.9772	0.9772	0.9772
NP+5mM IFN-γ		1	0.6904	0.9772	0.9772	0.9772	0.9772	0.9772
NP+50mM IFN-γ			1	0.6904	0.9181	0.6904	0.7731	0.6904
NP+100mM IFN-γ				1	0.9772	0.9772	0.9772	0.9772
P+0mM IFN-γ					1	0.9772	0.9772	0.9772
P+5mM IFN-γ						1	0.9772	0.9772
P+50mM IFN-γ							1	0.9772
P+100mM IFN-γ								1

	NP+0mM IL-10	NP+5mM IL-10	NP+50mM IL-10	NP+100mM IL-10	P+0mM IL-10	P+5mM IL-10	P+50mM IL-10	P+100mM IL-10
NP+0mM IL-10	1	0.9563	0.9563	0.9563	0.9563	0.9563	0.9563	0.9563
NP+5mM IL-10		1	0.9563	0.9563	0.9563	0.9563	0.9563	0.9563
NP+50mM IL-10			1	0.9563	0.9563	0.9563	0.9563	0.9563
NP+100mM IL-10				1	0.9563	0.9563	0.9563	0.9563
P+0mM IL-10					1	0.9563	0.9563	0.9563
P+5mM IL-10						1	0.9563	0.9563
P+50mM IL-10							1	0.9563
P+100mM IL-10								1

S6 Table (Cont.). Differences in cytokine expression due to K+ concentration and presence or absence of dental plaque. Kruskal-Wallis analysis corrected for multiple comparisons. In yellow are comparisons that were statistically significant with corrected p-value < 0.05. In green are comparisons that were statistically significant with corrected p-value < 0.1. NP, no plaque. P, plaque.

	NP+0mM IL-17A	NP+5mM IL-17A	NP+50mM IL-17A	NP+100mM IL-17A	P+0mM IL-17A	P+5mM IL-17A	P+50mM IL-17A	P+100mM IL-17A
NP+0mM IL-17A	1	0.9750	0.3072	0.9750	0.3072	0.9750	0.3072	0.9750
NP+5mM IL-17A		1	0.4013	0.9750	0.4141	0.9750	0.3072	0.9750
NP+50mM IL-17A			1	0.3072	0.9750	0.3072	0.9750	0.3072
NP+100mM IL-17A				1	0.3072	0.9750	0.3072	0.9750
P+0mM IL-17A					1	0.3160	0.9750	0.3072
P+5mM IL-17A						1	0.3072	0.9750
P+50mM IL-17A							1	0.3072
P+100mM IL-17A								1

	NP+0mM IL-1β	NP+5mM IL-1β	NP+50mM IL-1β	NP+100mM IL-1β	P+0mM IL-1β	P+5mM IL-1β	P+50mM IL-1β	P+100mM IL-1β
NP+0mM IL-1β	1	0.7358	0.7358	0.9013	0.9892	0.7358	0.6827	0.9892
NP+5mM IL-1β		1	0.6827	0.8868	0.7358	0.9892	0.6656	0.7358
NP+50mM IL-1β			1	0.7358	0.7358	0.6827	0.8323	0.7358
NP+100mM IL-1β				1	0.9013	0.8736	0.6827	0.9013
P+0mM IL-1β					1	0.7358	0.6827	1
P+5mM IL-1β						1	0.6656	0.7358
P+50mM IL-1β							1	0.6827
P+100mM IL-1β								1

S6 Table (Cont.). Differences in cytokine expression due to K+ concentration and presence or absence of dental plaque. Kruskal-Wallis analysis corrected for multiple comparisons. In yellow are comparisons that were statistically significant with corrected p-value < 0.05. In green are comparisons that were statistically significant with corrected p-value < 0.1. NP, no plaque. P, plaque.

	NP+0mM IL-6	NP+5mM IL-6	NP+50mM IL-6	NP+100mM IL-6	P+0mM IL-6	P+5mM IL-6	P+50mM IL-6	P+100mM IL-6
NP+0mM IL-6	1	0.4402	0.0608	0.0026	0.1040	0.0361	0.4402	0.0088
NP+5mM IL-6		1	0.2003	0.0055	0.2957	0.1260	1	0.0341
NP+50mM IL-6			1	0.0562	0.7479	0.0951	0.2003	0.2957
NP+100mM IL-6				1	0.0341	0.0951	0.0055	0.2957
P+0mM IL-6					1	0.5354	0.2957	0.1936
P+5mM IL-6						1	0.1260	0.4234
P+50mM IL-6							1	0.0341
P+100mM IL-6								1

	NP+0mM TNF-α	NP+5mM TNF-α	NP+50mM TNF-α	NP+100mM TNF-α	P+0mM TNF-α	P+5mM TNF-α	P+50mM TNF-α	P+100mM TNF-α
NP+0mM TNF-α	1	0.7727	0.0005	0.0000	0.8562	0.0086	0.0026	0.0000
NP+5mM TNF-α		1	0.0009	0.0000	0.8562	0.0044	0.0044	0.0000
NP+50mM TNF-α			1	0.0729	0.0006	0.0000	0.4155	0.0033
NP+100mM TNF-α				1	0.0000	0.0000	0.0121	0.1365
P+0mM TNF-α					1	0.0061	0.0033	0.0000
P+5mM TNF-α						1	0.0000	0.0000
P+50mM TNF-α							1	0.0006
P+100mM TNF-α								1
