# Supplementary material for: Potassium is a key signal in host-microbiome dysbiosis in periodontitis
Source: PLoS Pathog. 2017 Jun 20;13(6):e1006457. doi: 10.1371/journal.ppat.1006457 (PMC5493431; doi:10.1371/journal.ppat.1006457)
Supplement: S7 Table — A) IL-6 and TNF-α expression differences due to the addition of dental plaque. Kruskal-Wallis analysis corrected for multiple comparisons. In yellow are comparisons that were statistically significant with corrected p-value < 0.05. B) IL-6 and TNF-α expression differences due to K+ concentration regardless of the addition of dental plaque. Kruskal-Wallis analysis corrected for multiple comparisons. In yellow are comparisons that were statistically significant with corrected p-value < 0.05. C) IL-6 and TNF-α expression differences at high (50-100mM) and low (0-5mM) K+ concentration regardless of the addition of dental plaque. Kruskal-Wallis analysis corrected for multiple comparisons. In yellow are comparisons that were statistically significant with corrected p-value < 0.05. (PDF) [file ppat.1006457.s015.pdf]

S7 Table A. IL-6 and TNF-α expression differences due to the addition of dental plaque. Kruskal-Wallis analysis corrected for multiple comparisons. In yellow are comparisons that were statistically significant with corrected p-value < 0.05.

**IL-6**				
	Plaque 0mM	Plaque 5mM	Plaque 50mM	Plaque 100mM
No Plaque 0mM	0.1040			
No Plaque 5mM		0.1260		
No Plaque 50mM			0.2003	
No Plaque 100mM				0.2957
**TNF-α**				
No Plaque 0mM	0.8562			
No Plaque 5mM		0.0044		
No Plaque 50mM			0.4155	
No Plaque 100mM				0.1365

S7 Table B. IL-6 and TNF-α expression differences due to K^+ concentration regardless of the addition of dental plaque. Kruskal-Wallis analysis corrected for multiple comparisons. In yellow are comparisons that were statistically significant with corrected p-value < 0.05.

**IL-6**				
	0mM	5mM	50mM	100mM
0mM	1	0.5232	0.5571	0.0037
5mM		1	0.8333	0.0115
50mM			1	0.0106
100mM				1
**TNF-α**				
0mM	1	0.1145	0.0002	0.0000
5mM		1	0.0000	0.0000
50mM			1	0.0016
100mM				1
