# Supplementary material for: Potassium is a key signal in host-microbiome dysbiosis in periodontitis
Source: PLoS Pathog. 2017 Jun 20;13(6):e1006457. doi: 10.1371/journal.ppat.1006457 (PMC5493431; doi:10.1371/journal.ppat.1006457)
Supplement: S8 Table — See Fig 6. In yellow are comparisons of the effects of the different concentrations of K+ on hBD-3 expresssion that were statistically significant. (PDF) [file ppat.1006457.s016.pdf]

S8 Table. Kruskal-Wallis analysis corrected for multiple comparisons for expression levels of hBD-3. See Figure 6. In yellow are comparisons that were considered statistically significant (p-value < 0.05).

	Negative control	No Plaque 0mM	No Plaque 5mM	No Plaque 50mM	Plaque 0mM	Plaque 5mM	Plaque 50mM
Negative control		0	0	0	0	0	0
No Plaque 0mM			0	0.0113	0.0021	0	0
No Plaque 5mM				0.0002	0.0112	0.0113	0.6420
No Plaque 50mM					0.3318	0	0
Plaque 0mM						0	0.0020
Plaque 5mM							0.0198
Plaque 50mM							
