# Supplementary material for: Potassium is a key signal in host-microbiome dysbiosis in periodontitis
Source: PLoS Pathog. 2017 Jun 20;13(6):e1006457. doi: 10.1371/journal.ppat.1006457 (PMC5493431; doi:10.1371/journal.ppat.1006457)
Supplement: S9 Table — Output of the two-way ANOVA analysis to assess for significant interactions in expression levels of IL-6 and TNF-α. See S6 Fig. In yellow are comparisons that were statistically significant. (PDF) [file ppat.1006457.s017.pdf]

S9 Table. Analysis of Variance Tables. Output of the two-way ANOVA analysis to assess for significant interactions in expression levels of IL-6 and TNF-α. See S6 Fig. In yellow are comparisons that were statistically significant.

IL-6

Response:value						
	Df	Sum Sq	Mean Sq	F-value	Pr(>F)	
Presence of plaque	1	0.150	0.150	0.0176	0.8961168	
K^+ concentration	3	239.146	79.715	9.3266	0.0008424	***
Presence of plaque:K^+ concentration	3	98.895	32.965	3.8568	0.0298345	*
Residuals	16	136.754	8.547			

TNF-α

Response:value						
	Df	Sum Sq	Mean Sq	F-value	Pr(>F)	
Presence of plaque	1	50.1	50.14	3.7080	0.072113	.
K^+ concentration	3	3834.3	1278.09	94.5164	2.159e-10	***
Presence of plaque:K^+ concentration	3	349.0	116.34	8.6038	0.001247	**
Residuals	16	216.4	13.52			

Significance codes: 0 ‘***’ 0.001 ‘**’ 0.01 ‘*’ 0.05 ‘.’ 0.1
