# Supplementary material for: Potassium is a key signal in host-microbiome dysbiosis in periodontitis
Source: PLoS Pathog. 2017 Jun 20;13(6):e1006457. doi: 10.1371/journal.ppat.1006457 (PMC5493431; doi:10.1371/journal.ppat.1006457)
Supplement: S10 Table — Output of the two-way ANOVA analysis to assess for significant interactions in expression levels of hBD-3. See Fig 6. In yellow are comparisons that were statistically significant. (PDF) [file ppat.1006457.s018.pdf]

S10 Table. Analysis of Variance Table. Output of the two-way ANOVA analysis to assess for significant interactions in expression levels of hBD-3. See Figure 6. In yellow are comparisons that were statistically significant.

Response:value						
	Df	Sum Sq	Mean Sq	F	value	Pr(>F)
Presence of plaque	1	23.476	23.4758	31.3052	2.254e-07	***
K+ concentration	2	34.776	17.3879	23.1869	7.011e-09	***
Presence of plaque: K+ concentration	2	0.624	0.3118	0.4158	0.661	
Residuals	92	68.991	0.7499			
Signif. Codes: 0 ‘***’ ; 0.001 ‘**’ ; 0.01 ‘*’ ; 0.05						
